# Hydrogen Sulfide Prevents LPS-Induced Depression-like Behavior through the Suppression of NLRP3 Inflammasome and Pyroptosis and the Improvement of Mitochondrial Function in the Hippocampus of Mice

**DOI:** 10.3390/biology12081092

**Published:** 2023-08-05

**Authors:** Peng Bao, Yuxiang Gong, Yanjie Wang, Miaomiao Xu, Zhenyu Qian, Xin Ni, Jianqiang Lu

**Affiliations:** 1 School of Kinesiology, Shanghai University of Sport, Shanghai 200438, China; 2 National Clinical Research Center for Geriatric Disorders, Central South University Xiangya Hospital, Changsha 410008, China; 3 International Collaborative Research Center for Medical Metabolomics, Central South University Xiangya Hospital, Changsha 410008, China

**Keywords:** hydrogen sulfide, depression, NLRP3 inflammasome, pyroptosis, mitochondria

## Abstract

**Simple Summary:**

Depression is a significant public health problem, and its pathogenesis is associated with inflammation in the central nervous system. In this study, we investigated the effects of H_2_S donor NaHS treatment on depression-like behavior caused by lipopolysaccharide (LPS) and its potential mechanisms. It was found that H_2_S treatment prevented LPS-induced depression-like behavior. LPS resulted in NF-κB and NLRP3 inflammasome activation and pyroptosis in the hippocampus and led to hippocampal mitochondrial dysfunction, which could be reversed with H_2_S treatment. Our data indicate that H_2_S prevents LPS-induced depression-like behaviors via the inhibition of neuroinflammation and pyroptosis and improving mitochondrial function. H_2_S could be a promising therapeutic reagent for depression.

**Abstract:**

Hydrogen sulfide (H_2_S) has been implicated to have antidepressive effects. We sought to investigate the prevention effects of H_2_S donor NaHS on depression-like behavior induced by lipopolysaccharide (LPS) in mice and its potential mechanisms. Sucrose preference, force swimming, open field, and elevate zero maze were used to evaluate depression-like behavior. NF-κB and NLRP3 inflammasome activation and mitochondrial function in the hippocampus were determined. It was found that depression-like behavior induced by LPS was prevented by NaHS pretreatment. LPS caused NF-κB and NLRP3 inflammasome activation in the hippocampus as evidenced by increased phosphorylated-p65 levels and increased NLRP3, ASC, caspase-1, and mature IL-1β levels in the hippocampus, which were also blocked by NaHS. LPS increased GSDMD-N levels and TUNEL-positive cells in the hippocampus, which was prevented by NaHS. Abnormal mitochondrial morphology in the hippocampus was found in LPS-treated mice. Mitochondrial membrane potential and ATP production were reduced, and ROS production was increased in the hippocampus of LPS-treated mice. NaHS pretreatment improved impaired mitochondrial morphology and increased membrane potential and ATP production and reduced ROS production in the hippocampus of LPS-treated mice. Our data indicate that H_2_S prevents LPS-induced depression-like behaviors by inhibiting NLRP3 inflammasome activation and pyroptosis and improving mitochondrial function in the hippocampus.

## 1. Introduction

Major depressive disorder (MDD) is a kind of widespread psychological disorder characterized by a high incidence, high recurrence rate, low awareness rate, and high suicide mortality rate [1]. So far, the pathogenesis of depression is still unclear. However, many theories have been developed, such as imbalances of monoaminergic neurotransmitters, impaired hippocampal neurogenesis, mitochondrial dysfunction, and neuroinflammation, etc. [2,3].

Of note, it has been implicated that neuroinflammation would play a crucial role in the onset of depressive symptoms in certain instances [4]. Elevated levels of proinflammatory cytokines, including interleukin (IL)-1β, IL-6, and interferon-γ (IFN-γ), were found in cerebrospinal fluid and peripheral circulation in certain patients with MDD. Numerous studies reveal that inflammation, especially in chronic unexpected mild stress (CUMS)-induced [5,6] and LPS-induced models [7,8,9], aggravates the development of depressive-like behavior in certain brain regions. In the ovariectomized (OVX) model, elevated concentrations of the proinflammatory cytokines IL-6 and IFN-γ, as well as activated NF-κB, are linked to depressive-like behavior in the hippocampus [10]. Thus, the inhibition of neuroinflammation would be a promising therapeutic strategy for MDD.

Nucleotide-binding oligomerization domain-like receptor protein 3 (NLRP3) is a cytoplasmic pattern-recognition receptor (PRR) that undergoes self-oligomerization. It recruits apoptosis-associated speck-like proteins containing a caspase recruitment domain (ASC) and then combines with pro-caspase-1 to form the NLRP3/ASC/pro-caspase-1 protein complex, thereby promoting IL-1β and IL-18 maturation and gasdermin D (GSDMD)-dependent pyroptosis [11]. In patients with MDD, NLRP3 inflammasome is increased in mononuclear cells in peripheral circulation [12]. Our previous study found that NLRP3 inflammasome activation causes depression-like behaviors in the hippocampus of OVX mice [10]. Some studies have shown that NLRP3 inflammasome is activated in some other brain regions in LPS- and CUMS-induced depression models [13,14].

An increasing body of evidence shows that mitochondrial dysfunction is a significantly critical step in the onset and progression of depression [15]. Changes in mitochondrial function and morphology are revealed in the brains of patients with MDD [16]. Various studies have indicated that mitochondrial dysfunction may lead to depression-like behavior induced by LPS, chronic stress, and prenatal stress in rodent models [15,17,18,19,20,21]. In fact, abnormal mitochondrial function can lead to inflammation, and vice versa. The interplay between mitochondrial dysfunction and inflammation has been implicated in MDD development [22,23].

Hydrogen sulfide (H_2_S) is a variety of newfound gas-signaling molecule and has many biological functions including vasodilation, angiogenesis, cytoprotection, and anti-inflammation [24]. Some studies have shown that H_2_S can ameliorate LPS-induced and CUMS-induced depression-like behavior by suppressing microglia activation and endoplasmic reticulum stress, respectively [25,26,27,28,29]. However, whether H_2_S alleviation of depression-like behavior is associated with restraining the interplay between mitochondria dysfunction and inflammation remains to be elucidated.

The purpose of the present study was to investigate mechanisms underlying H_2_S prevention of depression-like behavior and to examine whether NLRP3 inflammasome and mitochondrial function contribute to it. To achieve this, we firstly observed the prevention effects of H_2_S donor NaHS on LPS-induced depression-like behavior and inflammation in the hippocampus of mice, and then examined mitochondrial function and NLRP3 inflammasome activation in the hippocampus in response to LPS and NaHS pretreatment.

## 2. Materials and Methods

### 2.1. Animals and Treatment

ICR mice weighing about 40 g (male, 8 weeks of age) were obtained from Gem Pharmatech (Nanjing, China). Animals were maintained in an SPF animal room at a temperature of 22 ± 2 °C with a 12 h light–dark cycle. Animals were provided with a standard pellet diet and water ab libitum. All animal experiments were approved by the Ethical Committee of Experimental Animals of the Shanghai University of Sport.

The mice were randomly divided into the following three groups, control group, LPS group, and LPS + NaHS group (*n* = 10 in each group). All animals were allowed to habituate for seven days before the following experiment. The mice of the LPS + NaHS group received an intraperitoneal (i.p.) injection of NaHS (5.6 mg/kg) for seven days. On the last day, the mice were also treated with LPS (0.83 mg/kg, ip). The mice of the LPS group were administrated with saline (i.p) for 6 days, and on the seventh day, the animals were injected with LPS (0.83 mg/kg, ip). Control mice received saline treatment for seven days. LPS (Escherichia coli 0111: B4) and NaHS were purchased from Sigma-Aldrich (Livonia, MI, USA) and dissolved in saline. The dosage of LPS and NaHS were chosen according to the literature [8,18,25,30]. Twenty-four hours after the last injection, the animals underwent behavior tests. After the behavior tests, the mice were firstly anesthetized with isoflurane and then cervical dislocation was quickly performed. The bilateral hippocampal tissues were quickly dissected from the brains. For Western blotting analysis, all samples were stored at −80 °C until analysis. For mitochondrial function, experiments were performed as soon as possible to keep the samples fresh. For TUNEL and immunofluorescence (IF) analysis, the mice were perfused with 4% paraformaldehyde (PFA) at the apex of the heart. After a series of procedures, the extracted brains were embedded with OCT compound. Then, serial slices of the hippocampus were cryosectioned (30 μm per slice) along the sagittal axis across the hippocampus using the freezing microtome (Leica, Wetzlar, Germany). All sections were frozen at −20 °C until analysis.

### 2.2. Behavior Analysis

All behavioral tests were conducted during 9:00–13:00 for mice. Before measurements, the mice were acclimated in the behavioral laboratory for at least two hours. In each test, animal behavior was recorded and analyzed using ANY-maze™ tracking software (Stoelting Co., Wooddale, IL, USA).

#### 2.2.1. Sucrose Preference Test (SPT)

Mice were single-housed and habituated with 1% sucrose solution for 3 days before the test [30]. Briefly, 72 h–48 h before the test, two bottles (50 mL tubes with stoppers) of 1% sucrose solution were placed in every cage for 24 h. At 48 h–24 h before the test, one bottle of 1% sucrose solution and one bottle of water were placed in every cage. Then, the mice were deprived of water and food for 24 h, followed by the test. The experimental animals had free access to two bottles containing 1% sucrose solution and water, respectively. The position of the bottles was interchanged every 12 h. After 24 h, the results were calculated with the following formula: percentage of sucrose preference = the weight of sucrose water consumption/the weight of total liquid intake.

#### 2.2.2. Forced Swimming Test (FST)

As previously mentioned, this test was conducted [31]. Briefly, each mouse was put into a transparent cylinder filled with 24 °C water. The test lasted 6 min and was divided into a pretest (the first 2 min) and a test (the last 4 min). Finally, the immobile time was recorded.

#### 2.2.3. Open Field Test (OFT)

The test was performed as described previously [32]. Briefly, each mouse was placed into the center of mental box (50 cm × 50 cm × 50 cm). The area ratio of the central zone to the peripheral zone was 9:25. Every session lasted for 6 min divided into pretest (the first 1 min) and test (the last 5 min); 75% alcohol was sprayed uniformly after each test. Finally, the shuttle distance and the number of entrances to the central zone were recorded.

#### 2.2.4. Elevate Zero Maze Test (EZM)

The elevated zero maze consisted of two open arms and two closed arms, as described previously [33]. The maze was placed 50 cm above the floor. The camera was kept above the maze. Each mouse was placed in the same position and allowed to explore the maze for 6 min. Each session was divided into a pretest (the first 1 min) and a test (the last 5 min); 75% alcohol was sprayed uniformly when the test ended. Finally, the results were calculated with the formula: percentage of entering open arms = the number of entering open arms/(the number of entering open arms + the number of entering closed arms).

#### 2.2.5. Integrated Behavioral Z-Normalization

The z-score was calculated for each mouse by computing how many standard deviations σ a given observation X (percentage of sucrose preference for SPT, immobile time for FST, shuttle distance and the number of times entering the central zone for OFT, and percentage of times entering the open arms for EZM) was from the group mean (μ). Finally, the z-scores for depression were averaged within each test such that each test had the same weight [17,34].

### 2.3. Mitochondria Isolation and Measurement of Mitochondrial Function

Hippocampal mitochondria were isolated using Tissue Mitochondria Isolation Kit (C3606, Beyotime, Shanghai, China) as described previously [30]. Briefly, the fresh hippocampal samples were rapidly placed into the PBS solution and washed twice. The samples were then homogenated with reagent A. After centrifugation of 1000× *g* for 5 min at 4 °C, the supernatant was subjected to centrifugation at 11,000× *g* for 10 min at 4 °C. The supernatants were discarded, and mitochondrial pellets were collected.

The mitochondrial membrane potential (MMP) was evaluated using the enhanced MMP assay kit with JC-1 (C2003S, Beyotime, Shanghai, China). The ATP concentration was assessed using an enhanced ATP Assay Kit (S0027, Beyotime, Shanghai, China). The ROS level was measured by the MitoSOX™ Red mitochondrial superoxide indicator (M36008, Invitrogen, Cheshire, UK).

### 2.4. Mitochondrial Respirometry

The fresh hippocampus was gently homogenized in an ice-cold MiR05 respiration medium with a PBI shredder. Next, the mixed solution was added into chambers for high-resolution respirometry (Oroboros O2K, Oroboros Instruments, Innsbruck, Austria) [17,35]. The substrate-uncoupler-inhibitor-titration (SUIT-) protocol was performed at 37 °C and 360–380 μM oxygen concentration.

Basal respiration (no additives, Routine) was measured when respiration was stabilized. Next, pyruvate (2000 mM), malate (400 mM), glutamate (2000 mM), and ADP (500 mM) were added to determine the oxidative phosphorylation capacity of complex I (CI OXPHOS). Succinate (1000 mM), as the substrate of complex II, was then added to measure the OXPHOS of complex I and complex II (CI&CII OXPHOS). Subsequently, protonophore carbonyl cyanide-m-chlorophenylhydrazone (CCCP, 1.0 mM) was titrated to assess the maximal uncoupled respiratory capacity of the electron transfer system (ETS). After that, the CII-supported uncoupled respiratory function (CII ETS) was evaluated after adding rotenone (1 mM). Residual oxygen consumption (ROX) was detected after the titration of antimycin A (5 mM) and malonic acid (2000 mM).

### 2.5. Electron Microscopy

Fresh hippocampal tissues were fixed lasting 24 h with the 2.5% glutaraldehyde solution. Following dehydration, the tissue was post-fixed using osmium tetroxide, and embedded in epoxy resin. The samples were cut into 50 nm slices, re-stained with lead citrate and uranyl acetate, and examined using a Hitachi H-7700 TEM (Hitachi, Tokyo, Japan).

### 2.6. Western Blotting Analysis

RIPA buffer containing phosphatase and protease inhibitors were used to lyse hippocampal tissues. After ultrasonic grinding and centrifugation, the supernatant was used for protein quantification with the BCA protein assay kit. A 30 μg protein of each sample was loaded and separated by 10% or 12.5% SDS-PAGE and then transferred to polyvinylidene difluoride (PVDF) membranes (Millipore, Bedford, MA, USA). Subsequently, the PVDF membranes were blocked with 5% BSA solution, followed by incubation with various primary antibodies (Supplementary Table S1) at 4 °C overnight. Following the washing procedure, the membranes were incubated with secondary antibodies lasting 1 h at room temperature. The enhanced chemiluminescence (ECL) and an image analyzer (Tanon, Shanghai, China) were used to detect these blots. Finally, Image J software 1.53 (NIH, Bethesda, MD, USA) was utilized to quantify them.

### 2.7. TdT-Mediated dUTP Nick-End Labeling (TUNEL) Assay

Programmed death cells contain DNA fragments, so they were detected using TUNEL assay. Frozen sections of hippocampal tissue were perfused with 4% paraformaldehyde for fixation for 10 min. Next, freshly prepared membrane permeabilization solution and balancing buffer were transferred to the sections. Then, sections were stained with the TUNEL reaction mixture (G1502, Service, Wuhan, China) and incubated for 2 h in the dark at 37 °C. The fluorescence was quantified using fluorescence microscopy (Olympus, Tokyo, Japan).

### 2.8. Statistical Analysis

All the data were presented as the mean ± SD. The normal distribution was evaluated using a Shapiro–Wilk test. One-way ANOVA followed by a post hoc test were used to evaluate the statistical differences between groups using SPSS 25.0 (SPSS, Chicago, IL, USA). The statistical significance threshold was set as *p* < 0.05.

## 3. Results

### 3.1. NaHS Pretreatment Prevents LPS-Induced Depression- and Anxiety-like Behavior

**Figure 1:**
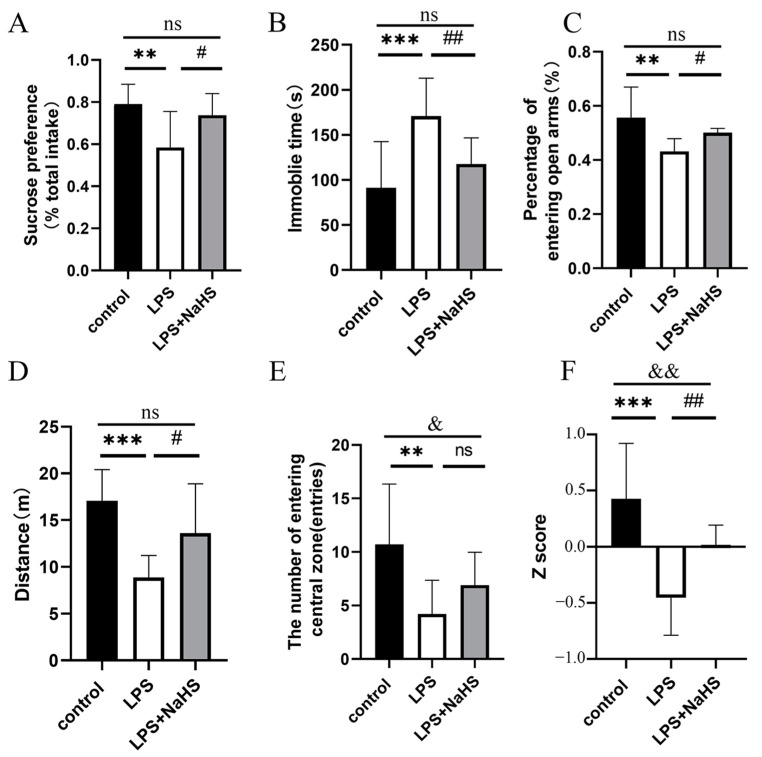
H_2_S attenuates depression-like and anxiety-like behavior caused by LPS in the mouse model. (**A**) Sucrose preference in SPT. (**B**) Immobile time in FST. (**C**) Percentage of number of entries in open arm in EZM. (**D**) Total distance traveled in OFT. (**E**) The number of times entering the central zone. (**F**) Integrated z-scores of depression across depression-related variables. All data are expressed as mean ± SD. ** *p* < 0.01, *** *p* < 0.001; ^#^ *p* < 0.05, ^##^ *p* < 0.01; ^&^ *p* < 0.05, ^&&^ *p* < 0.01; ns: no significance.

As shown in Figure 1, NaHS pretreatment efficiently ameliorated the effects of LPS on anhedonia and despair, which was evaluated using SPT and FST (Figure 1A,B). As for anxiety behavior, LPS treatment resulted in a decreased percentage of times entering the open arms in the EZM, reduced shuttle distance, and lowered time in the center zone in the OFT. NaHS significantly increased shuttle distance and did not affect the percentage of times entering the open arms in the EZM and time in the center zone in the OFT in LPS-treated mice (Figure 1C–E). Z-normalization analysis showed that the individual z-scores of mice pretreated with NaHS were significantly lower than those of mice treated without NaHS (Figure 1F), indicating an antidepressant effect of NaHS pretreatment.

### 3.2. H_2_S Pretreatment Alleviates NF-κB and NLRP3 Inflammasome Activation and Pyroptosis in the Hippocampus of LPS-Treated Mice

**Figure 2:**
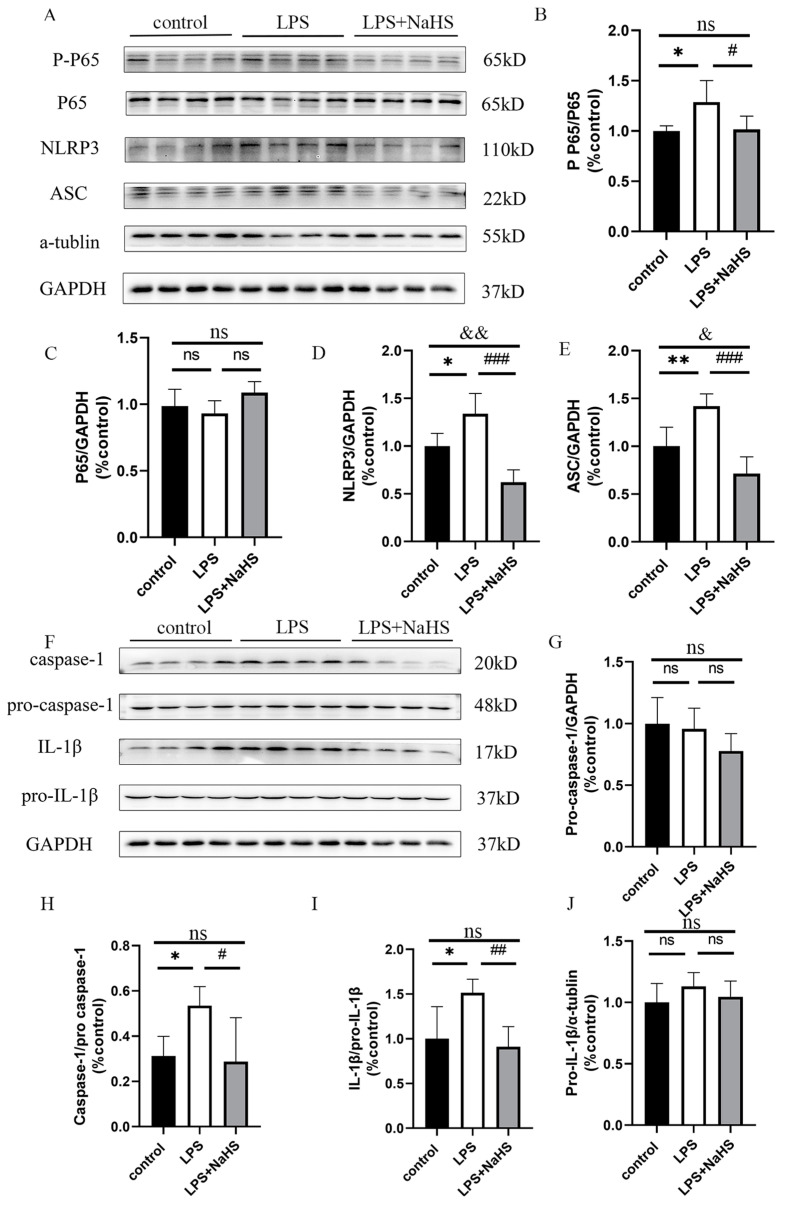
H_2_S prevents NF-κB and NLRP3 inflammasome activation in the hippocampus of LPS-treated mice. (**A**,**F**) Western blotting images of various proteins. (**B**–**E**,**G**–**J**) Quantitative diagrams of Western blot analysis. All data are expressed as mean ± SD. * *p* < 0.05, ** *p* < 0.01; ^#^ *p* < 0.05, ^##^ *p* < 0.01, ^###^ *p* < 0.001; ^&^ *p* < 0.05, ^&&^ *p* < 0.01; ns: no significance.

As shown in Figure 2A–C, LPS-induced NF-κB activation in the hippocampus as evidenced by increased p-p65 levels, which could be suppressed by NaHS pretreatment, suggesting that H_2_S prevented neuroinflammation in the hippocampus in LPS-treated mice. LPS treatment also induced NLRP3 inflammasome activation as evidenced by increased NLRP3, ASC, and caspase-1 levels, and IL-1β maturation, which is also prevented by NaHS pretreatment (Figure 2 and Supplemental Figures S1–S13). In addition, the levels of NLRP3 and ASC were lower in the NaHS + LPS group than those in the control group.

**Figure 3:**
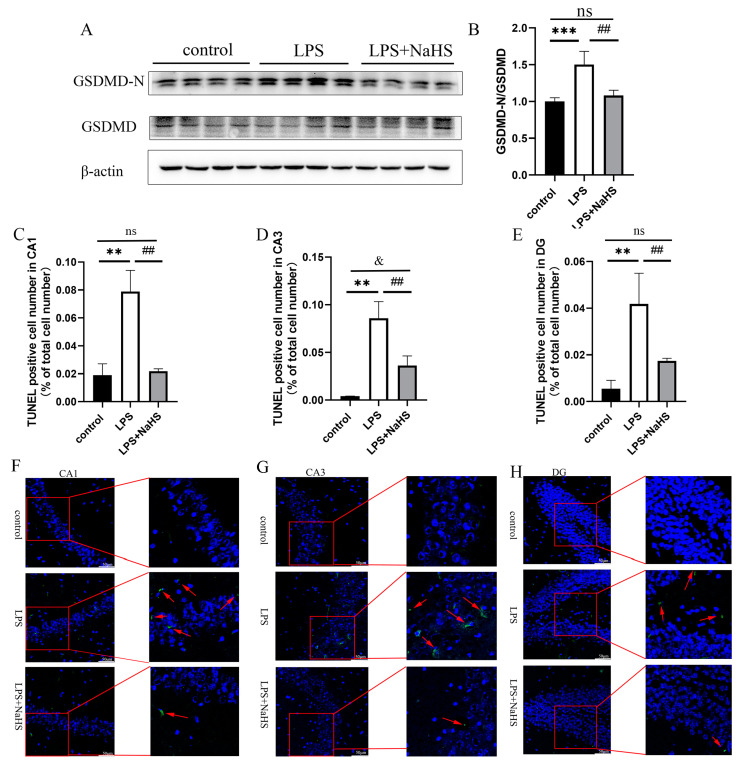
H_2_S suppresses pyroptosis in the hippocampus of LPS-treated mice. (**A**) Representative images of GSDMD-N and GSDMD blotting. (**B**) Quantitative diagram of Western blot analysis. (**C**–**E**) Percentage of TUNEL positive cells in CA1, CA3, and DG regions. (**F**–**H**) Representative images of TUNEL positive cells in CA1, CA3, and DG regions (×200). Red arrows indicate the TUNEL-positive cells. All data are expressed as mean ± SD. ** *p* < 0.01, *** *p* < 0.001; ^##^ *p* < 0.01; ^&^ *p* < 0.05, ns: no significance.

NLRP3 inflammasome activation also leads to the cleavage of full-length GSDMD to generate the GSDMD N-terminal, thereby leading to pyroptosis. An increase in the level of the GSDMD N-terminus was found in LPS-treated mice, which was significantly inhibited by NaHS pretreatment (Figure 3A,B and Supplemental Figures S14–S16). TUNEL staining showed that LPS increased the percentage of positive staining cells in the region of CA1, CA3, and DG, which also decreased in the NaHS pretreatment group, suggesting that NaHS prevents NLRP3 inflammasome activation and pyroptosis caused by LPS (Figure 3C–H).

### 3.3. H_2_S Prevents Mitochondrial Damage in the Hippocampus of LPS-Treated Mice

**Figure 4:**
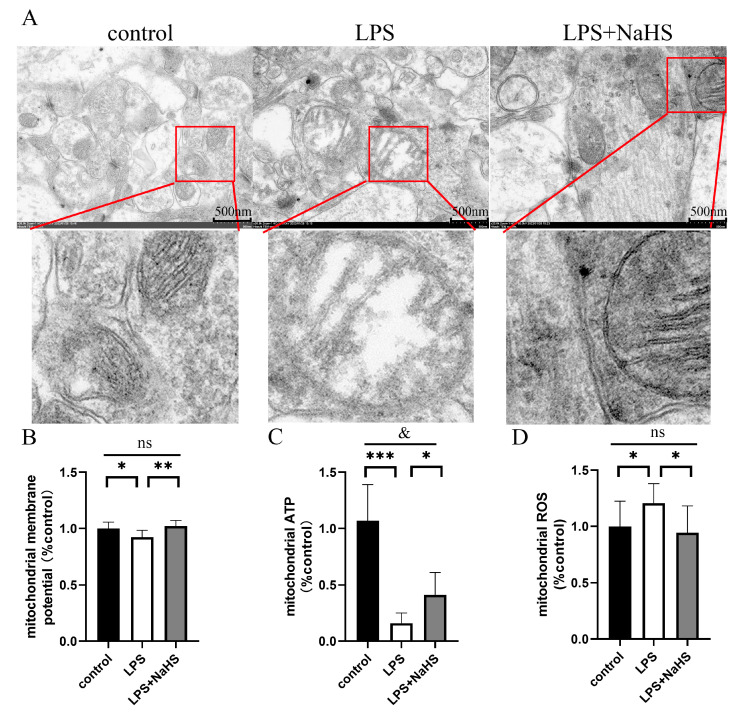
H_2_S improves mitochondrial morphology and function in the hippocampus of LPS-treated mice. (**A**) Representative TEM images of mitochondria in the hippocampus (×20 k). Red arrows indicate the local magnification of the mitochondria. (**B**–**D**) Mitochondrial function: membrane potential (**B**), ATP production (**C**), and ROS production (**D**). All data are expressed as mean ± SD. * *p* < 0.05, ** *p* < 0.01, *** *p* < 0.001; ^&^ *p* < 0.05, ns: no significance.

As shown in Figure 4A, LPS caused the mitochondrial swelling and blurring of ridges in hippocampal neurons, which could be improved by NaHS pretreatment. Consistently, LPS resulted in mitochondrial membrane potential, and ATP production was reduced, while ROS production was increased in the hippocampus models, which was significantly improved by NaHS pretreatment (Figure 4B–D).

**Figure 5:**
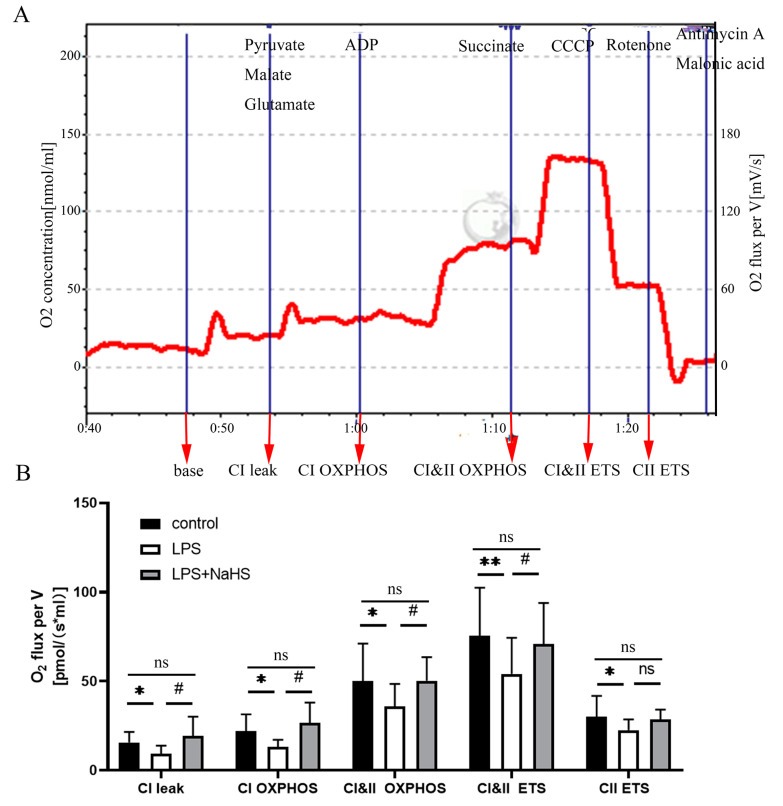
H_2_S improves the respiratory rate of mitochondrial complexes of the hippocampus in the LPS-induced depression model. (**A**) Representative tracing of the determination of OXPHOS complex I and II. Blue line: real-time oxygen consumption. Red line: real-time respiratory rate. (**B**) Statistical data of complex I and II. All data are expressed as mean ± SD. * *p* < 0.05, ** *p* < 0.01; ^#^ *p* < 0.05, ns: no significance.

Mitochondrial respirometry showed that CI leak, CI OXPHOS, CI&II OXPHOS, CI&II ETS, and CII ETS were significantly lower in the LPS treatment group than those of the control group. With the treatment of NaHS, the oxygen consumption rates for CI OXPHOS, CI&II OXPHOS, and CI&II ETS were obviously increased, but CII ETS was not significantly affected (Figure 5).

## 4. Discussion

The present study has shown that LPS could lead to depression-like behavior, which could be reversed by H_2_S treatment. Moreover, we have also demonstrated that NF-κB and NLRP3 inflammasome activation and pyroptosis as well as mitochondrial dysfunction in the hippocampus in mice, could be prevented by H_2_S treatment.

Many studies have proposed that sterile inflammation has a crucial role in the pathogenesis of depression [36,37,38]. Peripheral LPS treatment causes a systemic immune response, which eventually induces behavior deficits after 24 h [8]. As mentioned, the inflammatory responses occur in the central nervous system (CNS) in patients with MDD [39,40] and rodent models of depression, including the LPS-induced depression-like mouse model [37,41,42]. We also found that inflammation occurred consistently in the hippocampus of LPS-treated mice, and it was suppressed when the depression-like behavior was improved.

NLRP3 inflammasome was very crucial in the onset of various inflammatory responses [23]. As mentioned, NLRP3 inflammasome activation occurs in the CNS in the LPS-induced depression-like mouse model. The present study also revealed NLRP3 inflammasome activation in the hippocampus upon LPS treatment. It has also been shown that the blockage of NLRP3 inflammasome activation by specific NLRP3 inflammasome inhibitors could alleviate depression-like behavior in mice [7,10,41,43]. We consistently found that hippocampal NLRP3 inflammasome activation was prevented in LPS-treated mice with H_2_S pretreatment. It therefore indicates that the suppression of hippocampal NLRP3 inflammasome activation contributes to the H_2_S prevention of LPS-induced depression-like behavior.

Pyroptosis, a form of programmed necrosis, is linked to a general innate immune effector mechanism in psychological disorders [44,45]. It is known that NLRP3 inflammasome induces pyroptosis in various tissues [46]. The full-length GSDMD protein is cleaved to produce the GSDMD N-terminus as a result of NLRP3 inflammasome activation, which causes cell necrosis [37,44,47]. In this study, we found that pyroptosis occurred in the hippocampus of the LPS-induced model. Moreover, pyroptosis in the hippocampus was suppressed by H_2_S pretreatment. Interestingly, another study has also shown that H_2_S suppresses hypoxia-induced pyroptosis in the adrenal glands of mice [48]. Thus, our data indicate that hippocampal pyroptosis contributes to LPS-induced depression-like behavior and the suppression of pyroptosis is associated with the H_2_S prevention of depression-like behavior.

As mentioned, mitochondrial dysfunction plays a critical role in neuropsychiatric disorders including MDD [49]. LPS can induce mitochondrial abnormalities in the CNS of the LPS-induced depression-like mouse model. We consistently found that abnormal mitochondria morphology and function in the hippocampus of LPS-treated mice. Of note, there were a few studies on the effects of H_2_S on mitochondrial function in the CNS in depression-like models, although some studies have demonstrated that H_2_S can alleviate mitochondrial dysfunction in various tissues [50,51,52]. In the present study, we showed that H_2_S treatment reversed LPS-induced abnormal mitochondrial morphology and function in the hippocampus. Mitochondria can activate the NLRP3 inflammasome in two ways: in an ROS-dependent and ROS-independent manner. ROS can lead to producing oxidized mtDNA; the latter activates NLRP3 inflammasome. Three mitochondrial proteins, cardiolipin, mitochondrial antiviral signaling protein (MAVS), and mitofusin 2 (MFN 2), have been implicated in the activation of NLRP3 inflammasome in an ROS-independent manner [23]. It is well known that inflammation can cause mitochondrial dysfunction in various tissues [53,54]. Thus, our above findings that H_2_S pretreatment improves mitochondrial function and the suppression of NLRP3 inflammasome activation indicate that H_2_S arrests the interplay of mitochondrial dysfunction in the hippocampus of the LPS-induced depression-like model.

Many studies have demonstrated that H_2_S has therapeutic effects on depression-like behavior in various animal models of depression [25,26,27,28]. Of note, some studies also show that the endogenous H^2^S level in the brain is reduced in various rodent models of depression [26,28,29]. Kumar et al. [26] reported that the H2S level in the hippocampus is reduced upon LPS treatment, which contributes to depression-like behavior in mice. Thus, NaHS pretreatment could prevent a reduced H2S level in the hippocampus.

There are several limitations in the present study. In addition to the hippocampus, other brain regions such as the prefrontal cortex and amygdala also play an important role in depression pathogenesis [55,56,57]. It would be of interest to investigate the effects of H2S on these brain regions in depression-like models. Furthermore, the cell types responsible for NLRP3 inflammasome activation and mitochondrial dysfunction caused by LPS have not been clarified, since both microglia and neurons have been shown to play roles in LPS-induced depression-like behavior. Mitochondria are highly dynamic organelles, and their normal function depends on the regulation of the mitochondrial quality control system in depression [42]. This should also be investigated in future studies.

## 5. Conclusions

In conclusion, our study reveals that NLRP3 inflammasome activation and pyroptosis as well as abnormal mitochondrial morphology and function occur in the hippocampus of the LPS-induced depression-like mouse model. H_2_S pretreatment prevents LPS-induced depression-like behavior through the inhibition of neuroinflammation and pyroptosis and improving mitochondrial function in the hippocampus. Our study immediately highlights that H_2_S can be a promising prevention reagent for depression.

## Data Availability

Not applicable.

## Supplementary Materials

The following supporting information can be downloaded at: https://www.mdpi.com/article/10.3390/biology12081092/s1, Table S1: The antibodies employed in this study; Figure S1: Western blot membrane of P65 (~65 kDa) protein detected with anti-NF-κB (C22B4; 1:1000; CST, USA) antibody; Figure S2: Western blot membrane of P-P65 (~65 kDa) protein detected with anti-P-NF-κB (S536; 1:500; CST, USA) antibody; Figure S3: Western blot membrane of NLRP3 (~110 kDa) protein detected with anti-NLRP3 (ab214185; 1:1000; Abcam, UK) antibody; Figure S4: Western blot membrane of NLRP3 (~110 kDa) protein detected with anti-NLRP3 (ab214185; 1:1000; Abcam, UK) antibody; Figure S5: Western blot membrane of GAPDH (~37 kDa) protein detected with anti-GAPDH (MB001; 1:10000; Bioworld, USA) antibody; Figure S6: Western blot membrane of ASC (~22 kDa) protein detected with anti-ASC (A11433; 1:1000; ABclonal, China) antibody; Figure S7: Western blot membrane of GAPDH (~37 kDa) protein detected with anti-GAPDH (MB001; 1:10000; Bioworld, USA) antibody; Figure S8: Western blot membrane of pro-caspase-1 (~48 kDa) protein detected with anti-caspase-1 (A0964; 1:1000; ABclonal, China) antibody; Figure S9: Western blot membrane of caspase-1 (~20 kDa) protein detected with anti-caspase-1 (A0964; 1:1000; ABclonal, China) antibody; Figure S10: Western blot membrane of GAPDH (~37 kDa) protein detected with anti-GAPDH (MB001; 1:10000; Bioworld, USA) antibody; Figure S11: Western blot membrane of pro-IL-1β (~37 kDa) protein detected with anti-IL-1β (A11369; 1:1000; ABclonal, China) antibody; Figure S12: Western blot membrane of IL-1β (~17 kDa) protein detected with anti-IL-1β (A11369; 1:1000; ABclonal, China) antibody; Figure S13: Western blot membrane of α-tublin (~55 kDa) protein detected with anti-α-tublin (BS1699; 1:10000; Bioworld, USA) antibody; Figure S14: Western blot membrane of GSDMD (~53 kDa) protein detected with anti-GSDMD (A20197; 1:1000; ABclonal, China) antibody; Figure S15: Western blot membrane of GSDMD-N (~37 kDa) protein detected with anti-GSDMD-N (A20197; 1:1000; ABclonal, China) antibody; Figure S16: Western blot membrane of β-actin (~43 kDa) protein detected with anti-β-actin (BS6007M; 1:10000; Bioworld, USA) antibody.
