# Supplementary material for: Hydrogen Sulfide Prevents LPS-Induced Depression-like Behavior through the Suppression of NLRP3 Inflammasome and Pyroptosis and the Improvement of Mitochondrial Function in the Hippocampus of Mice
Source: Biology (Basel). 2023 Aug 5;12(8):1092. doi: 10.3390/biology12081092 (PMC10451782; doi:10.3390/biology12081092)
Supplement: Supplementary file 1 [file biology-12-01092-s001.zip › biology-2484834-supplementary.pdf]

Table S1. The antibodies employed in this study.

Antibodies	Source	Nation	Code number	Application
anti-NF-κB	CST	USA	C22B4	WB（1:1000）
anti-p-NF-κB	CST	USA	S536	WB（1:500）
anti-NLRP3	Abcam	UK	ab214185	WB（1:1000）
anti-ASC	ABclonal	China	A11433	WB（1:1000）
anti-IL-1β	ABclonal	China	A11369	WB（1:1000）
anti-Caspase-1	ABclonal	China	A0964	WB（1:1000）
anti-GSDMD	ABclonal	China	A20197	WB（1:1000）
anti-rabbit IgG	ABclonal	China	AS014	WB（1:1000）
anti-GAPDH	Bioworld	USA	MB001	WB（1:10000）
anti-α-tublin	Bioworld	USA	BS1699	WB（1:10000）
anti-β-actin	Bioworld	USA	BS6007M	WB（1:10000）

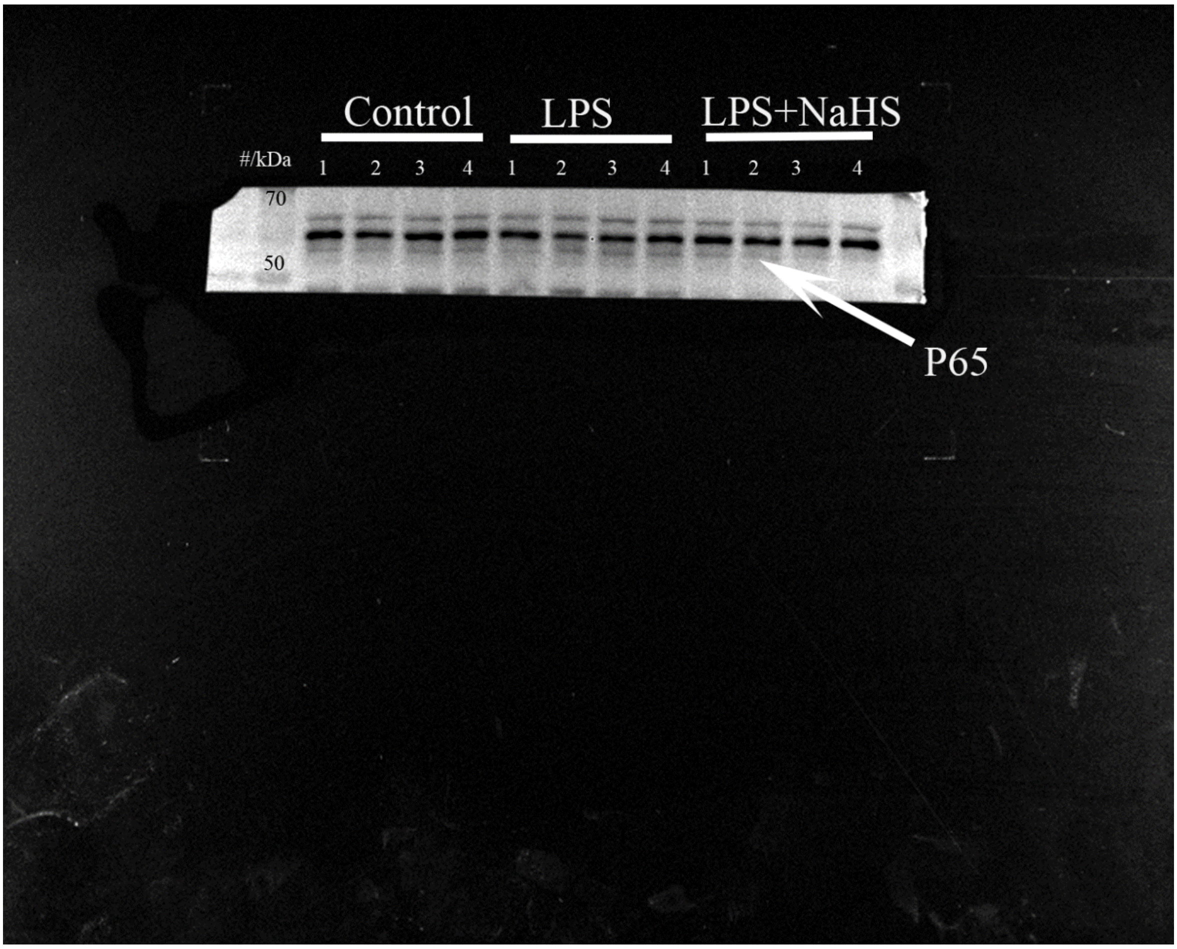

Figure S1. Western blot membrane of P65 (~65 kDa) protein detected with anti-NF-κB (C22B4; 1:1000; CST, USA) antibody. Gel-separated proteins were transferred to PVDF membranes (0.2 μm pore size; Millipore, Bedford, MA) via wet electroblotting (200mA, 60 min). Membranes, incubated with a horseradish peroxidase-conjugated secondary antibody (AS014; 1:1000; ABclonal), were developed with Omni-ECL™Femto Light Chemiluminescence Kit (Epizyme). #Weight marker (molecular weight in kDa): Multicolor Prestained Protein Ladder, 10 to 250 kDa; catalogue number: WJ102i. Blot images, prior to the densitometry readings, were converted to grayscale with ImageJ (ImageJ, National Institutes of Health, Maryland, USA) as follows: Image -> Type –> 8 bit. Next: Analyze->set scale->Click to remove scale. Then: Process->Subtract background->rolling ball radius 50 pixels-> light background. Final: Edit->invert.

Control 1, 2, 3, and 4 refer to four different samples from the control group.
LPS 1, 2, 3, and 4 show four different samples from the LPS group.
LPS+NaHS 1, 2, 3, and 4 indicate four different samples from the LPS+NaHS group.

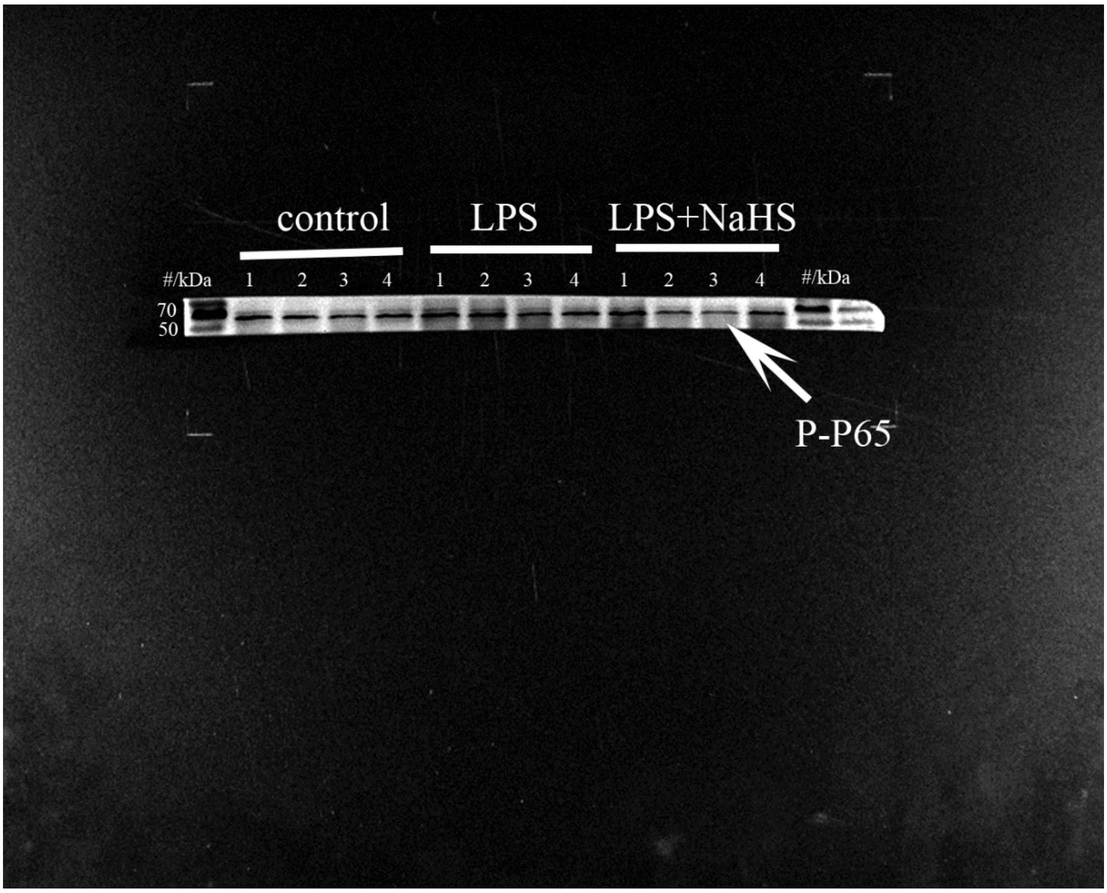

Figure S2. Western blot membrane of P-P65 (~65 kDa) protein detected with anti-P-NF-κB (S536; 1:500; CST, USA) antibody. Gel-separated proteins were transferred to PVDF membranes (0.2 μm pore size; Millipore, Bedford, MA) via wet electroblotting (200mA, 60 min). Membranes, incubated with a horseradish peroxidase-conjugated secondary antibody (AS014; 1:1000; ABclonal), were developed with Omni-ECL™Femto Light Chemiluminescence Kit (Epizyme). #Weight marker (molecular weight in kDa): Multicolor Prestained Protein Ladder, 10 to
250 kDa; catalogue number: WJ102i. Blot images, prior to the densitometry readings, were converted to grayscale with ImageJ (ImageJ, National Institutes of Health, Maryland, USA) as follows: Image -> Type –> 8 bit. Next: Analyze->set scale->Click to remove scale, Then: Process->Subtract background->rolling ball radius 50 pixels-> light background. Final: Edit->invert.

Control 1, 2, 3, and 4 refer to four different samples from the control group.
LPS 1, 2, 3, and 4 show four different samples from the LPS group.
LPS+NaHS 1, 2, 3, and 4 indicate four different samples from the LPS+NaHS group.

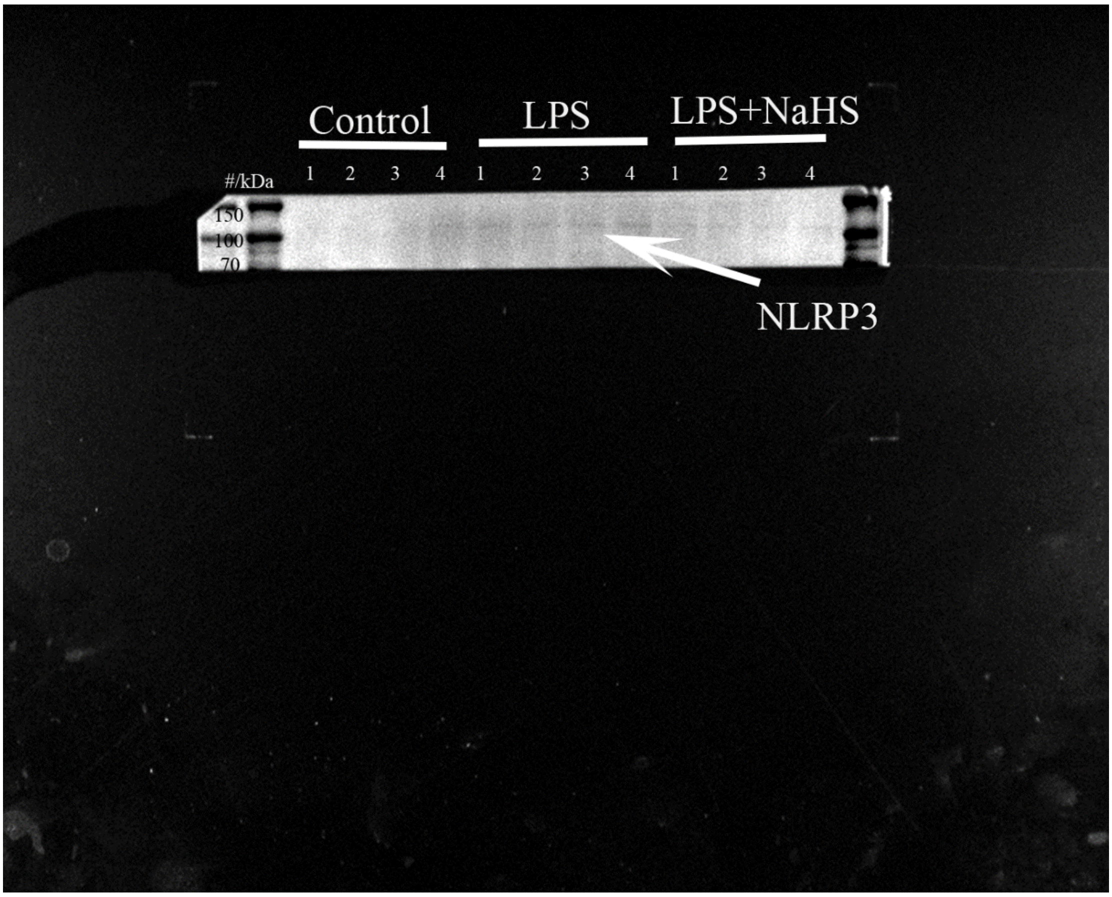

Figure S3. Western blot membrane of NLRP3 (~110 kDa) protein detected with anti-NLRP3 (ab214185; 1:1000; Abcam, UK) antibody. Gel-separated proteins were transferred to PVDF membranes (0.2 μm pore size; Millipore, Bedford, MA) by wet electroblotting (200mA, 60 min). Membranes, incubated with a horseradish peroxidase-conjugated secondary antibody (AS014; 1:1000; ABclonal), were developed with Omni-ECL™Femto Light Chemiluminescence Kit (Epizyme). #Weight marker (molecular weight in kDa): Multicolor Prestained Protein Ladder, 10 to 250 kDa; catalogue number: WJ102i. Blot images, prior to the densitometry readings, were converted to grayscale with ImageJ (ImageJ, National Institutes of Health, Maryland, USA) as follows: Image -> Type –> 8 bit. Next: Analyze->set scale->Click to remove scale. Then: Process->Subtract background->rolling ball radius 50 pixels-> light background. Final: Edit->invert.

Control 1, 2, 3, and 4 refer to four different samples from the control group.
LPS 1, 2, 3, and 4 show four different samples from the LPS group.
LPS+NaHS 1, 2, 3, and 4 indicate four different samples from the LPS+NaHS group.

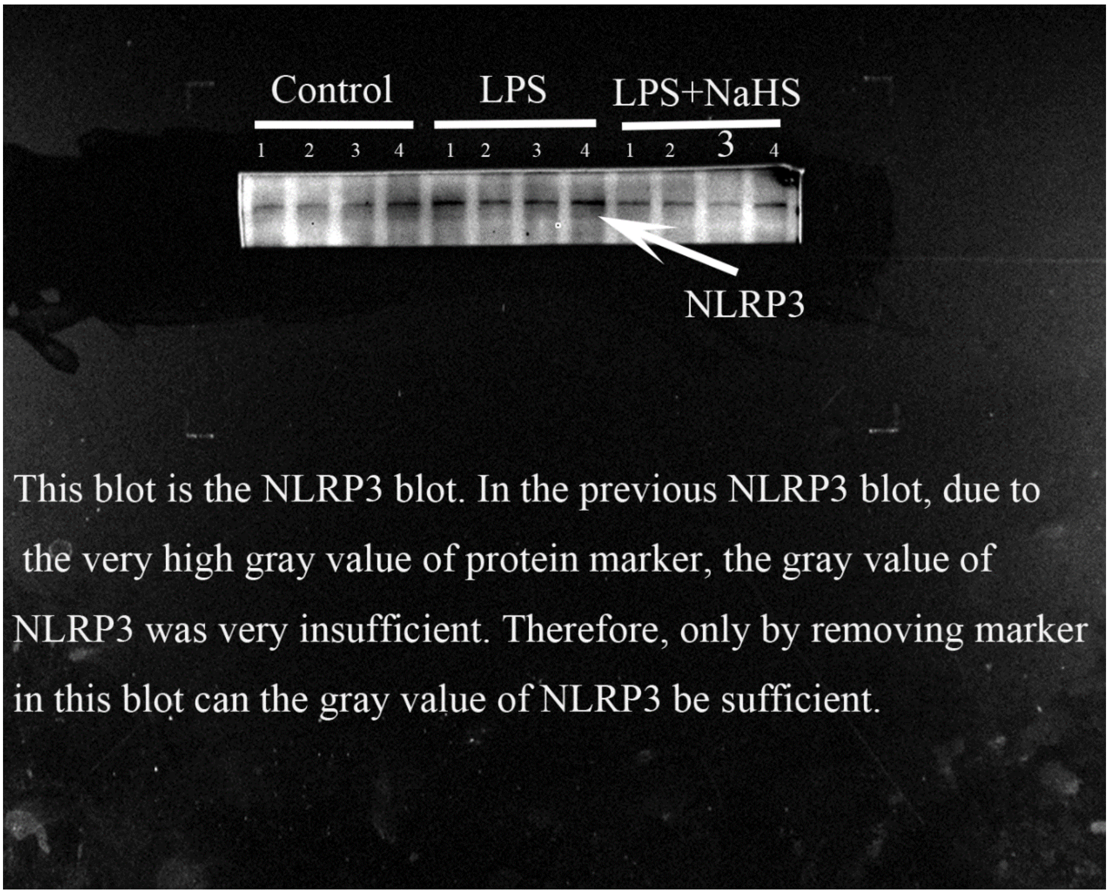

Figure S4. Western blot membrane of NLRP3 (~110 kDa) protein detected with anti-NLRP3 (ab214185; 1:1000; Abcam, UK) antibody. Gel-separated proteins were transferred to PVDF membranes (0.2 μm pore size; Millipore, Bedford, MA) via wet electroblotting (200mA, 60 min). Membranes, incubated with a horseradish peroxidase-conjugated secondary antibody (AS014; 1:1000; ABclonal), were developed with Omni-ECL™Femto Light Chemiluminescence Kit (Epizyme). #Weight marker (molecular weight in kDa): Multicolor Prestained Protein Ladder, 10 to 250 kDa; catalogue number: WJ102i. Blot images, prior to the densitometry readings, were converted to grayscale with ImageJ (ImageJ, National Institutes of Health, Maryland, USA) as follows: Image -> Type –> 8 bit. Next: Analyze->set scale->Click to remove scale. Then: Process->Subtract background->rolling ball radius 50 pixels-> light background. Final: Edit->invert.

Control 1, 2, 3, and 4 refer to four different samples from the control group.
LPS 1, 2, 3, and 4 show four different samples from the LPS group.
LPS+NaHS 1, 2, 3, and 4 indicate four different samples from the LPS+NaHS group.

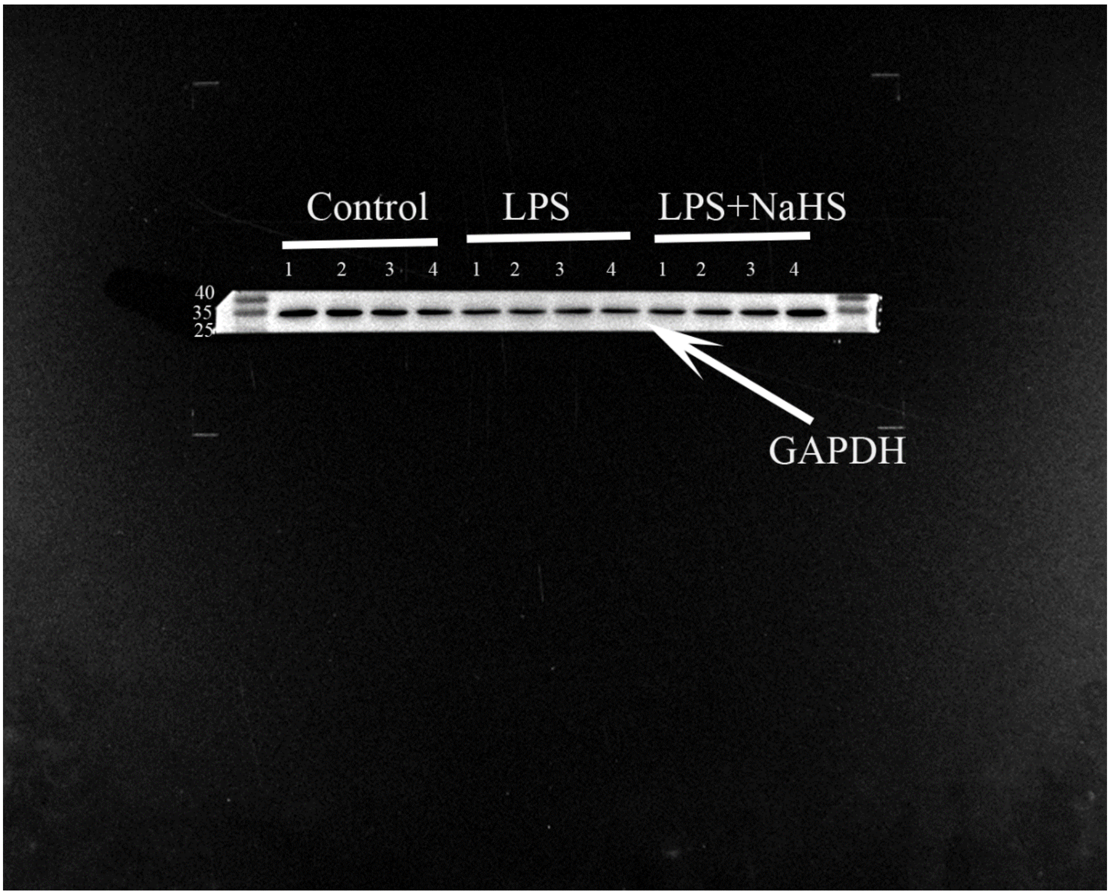

Figure S5. Western blot membrane of GAPDH (~37 kDa) protein detected with anti-GAPDH (MB001; 1:10000; Bioworld, USA) antibody. Gel-separated proteins were transferred to PVDF membranes (0.2 μm pore size; Millipore, Bedford, MA) via wet electroblotting (200mA, 60 min). Membranes, incubated with a horseradish peroxidase-conjugated secondary antibody (AS014; 1:1000; ABclonal), were developed with Omni-ECL™Femto Light Chemiluminescence Kit (Epizyme). #Weight marker (molecular weight in kDa): Multicolor Prestained Protein Ladder, 10 to 250 kDa; catalogue number: WJ102i. Blot images, prior to the densitometry readings, were converted to grayscale with ImageJ (ImageJ, National Institutes of Health, Maryland, USA) as follows: Image -> Type –> 8 bit. Next: Analyze->set scale->Click to remove scale. Then: Process->Subtract background->rolling ball radius 50 pixels-> light background. Final: Edit->invert.

Control 1, 2, 3, and 4 refer to four different samples from the control group.
LPS 1, 2, 3, and 4 show four different samples from the LPS group.
LPS+NaHS 1, 2, 3, and 4 indicate four different samples from the LPS+NaHS group.

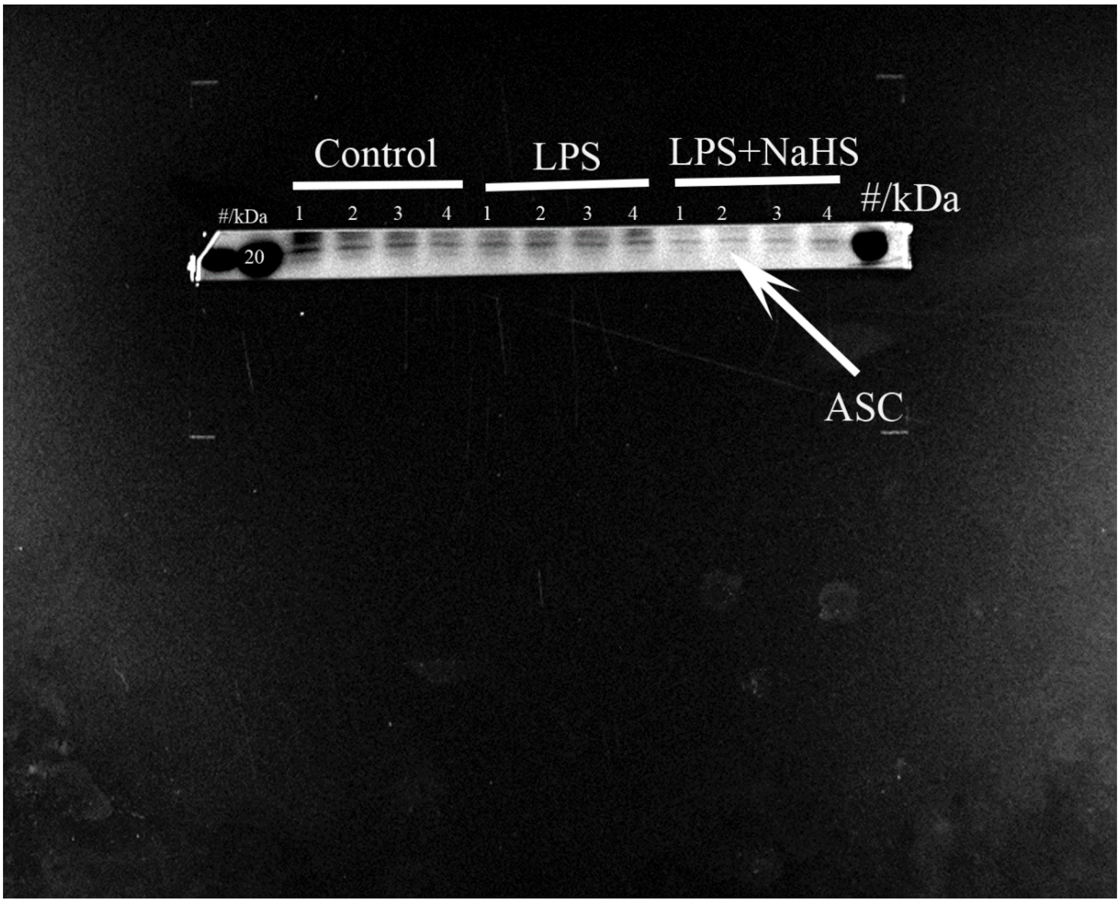

Figure S6. Western blot membrane of ASC (~22 kDa) protein detected with anti-ASC (A11433; 1:1000; ABclonal, China) antibody. Gel-separated proteins were transferred to PVDF membranes (0.2 μm pore size; Millipore, Bedford, MA) via wet electroblotting (200mA, 60 min). Membranes, incubated with a horseradish peroxidase-conjugated secondary antibody (AS014; 1:1000; ABclonal), were developed with Omni-ECL™Femto Light Chemiluminescence Kit (Epizyme). #Weight marker (molecular weight in kDa): Multicolor Prestained Protein Ladder, 10 to 250 kDa; catalogue number: WJ102i. Blot images, prior to the densitometry readings, were converted to grayscale with ImageJ (ImageJ, National Institutes of Health, Maryland, USA) as follows: Image -> Type –> 8 bit. Next: Analyze->set scale->Click to remove scale. Then: Process->Subtract background->rolling ball radius 50 pixels-> light background. Final: Edit->invert.

Control 1, 2, 3, and 4 refer to four different samples from the control group.
LPS 1, 2, 3, and 4 show four different samples from the LPS group.
LPS+NaHS 1, 2, 3, and 4 indicate four different samples from the LPS+NaHS group.

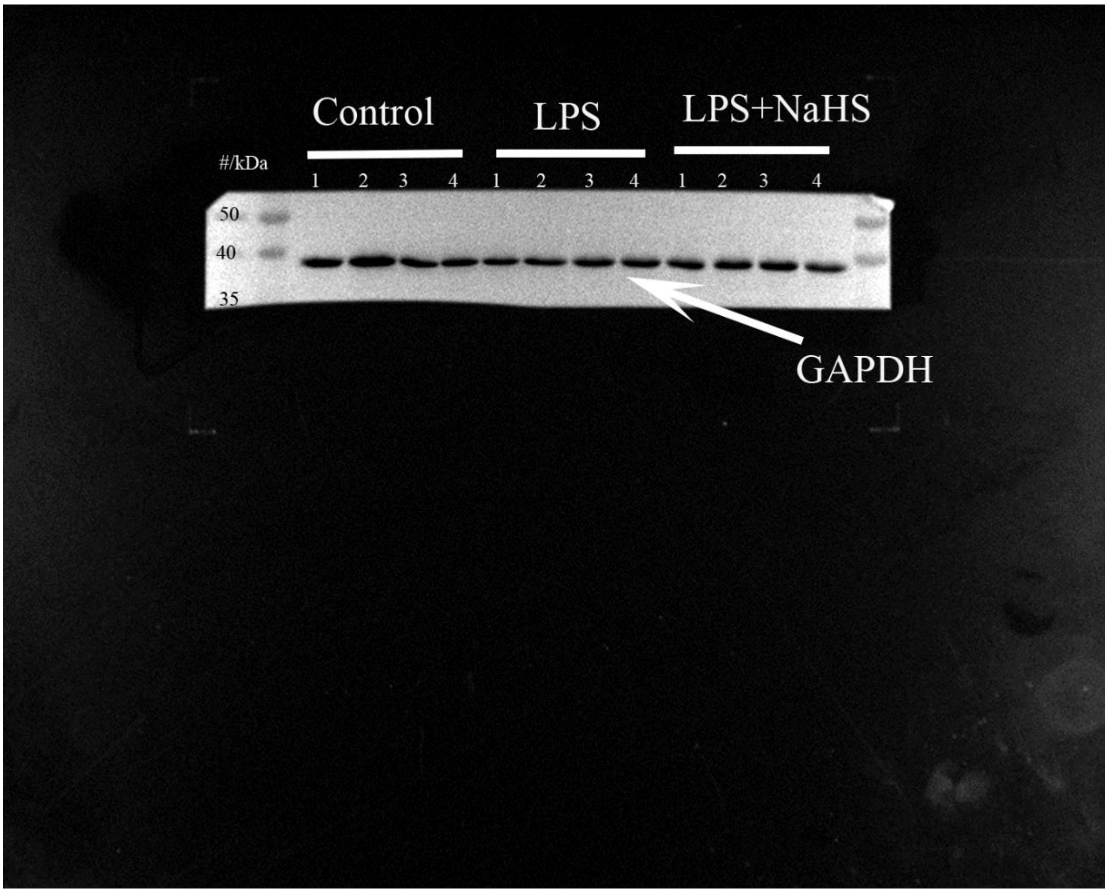

Figure S7. Western blot membrane of GAPDH (~37 kDa) protein detected with anti-GAPDH (MB001; 1:10000; Bioworld, USA) antibody. Gel-separated proteins were transferred to PVDF membranes (0.2 μm pore size; Millipore, Bedford, MA) via wet electroblotting (200mA, 60 min). Membranes, incubated with a horseradish peroxidase-conjugated secondary antibody (AS014; 1:1000; ABclonal), were developed with Omni-ECL™Femto Light Chemiluminescence Kit (Epizyme). #Weight marker (molecular weight in kDa): Multicolor Prestained Protein Ladder, 10 to 250 kDa; catalogue number: WJ102i. Blot images, prior to the densitometry readings, were converted to grayscale with ImageJ (ImageJ, National Institutes of Health, Maryland, USA) as follows: Image -> Type –> 8 bit. Next: Analyze->set scale->Click to remove scale. Then: Process->Subtract background->rolling ball radius 50 pixels-> light background. Final: Edit->invert.

Control 1, 2, 3, and 4 refer to four different samples from the control group.
LPS 1, 2, 3, and 4 show four different samples from the LPS group.
LPS+NaHS 1, 2, 3, and 4 indicate four different samples from the LPS+NaHS group.

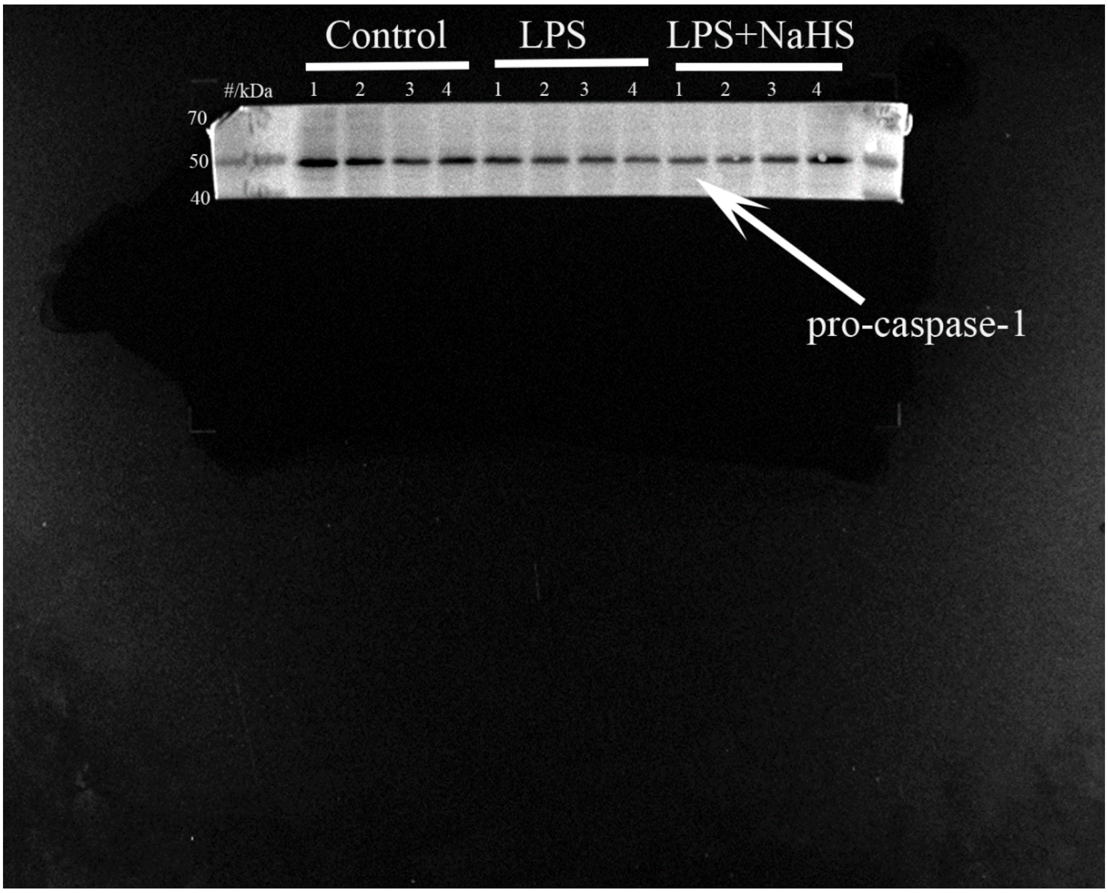

Figure S8. Western blot membrane of pro-caspase-1 (~48 kDa) protein detected with anti-caspase-1 (A0964; 1:1000; ABclonal, China) antibody. Gel-separated proteins were transferred to PVDF membranes (0.2 μm pore size; Millipore, Bedford, MA) via wet electroblotting (200mA, 30 min). Membranes, incubated with a horseradish peroxidase-conjugated secondary antibody (AS014; 1:1000; ABclonal), were developed with Omni-ECL™Femto Light Chemiluminescence Kit (Epizyme). #Weight marker (molecular weight in kDa): Multicolor Prestained Protein Ladder, 10 to 250 kDa; catalogue number: WJ102i. Blot images, prior to the densitometry readings, were converted to grayscale with ImageJ (ImageJ, National Institutes of Health, Maryland, USA) as follows: Image -> Type –> 8 bit. Next: Analyze->set scale->Click to remove scale. Then: Process->Subtract background->rolling ball radius 50 pixels-> light background. Final: Edit->invert.

Control 1, 2, 3, and 4 refer to four different samples from the control group.
LPS 1, 2, 3, and 4 show four different samples from the LPS group.
LPS+NaHS 1, 2, 3, and 4 indicate four different samples from the LPS+NaHS group.

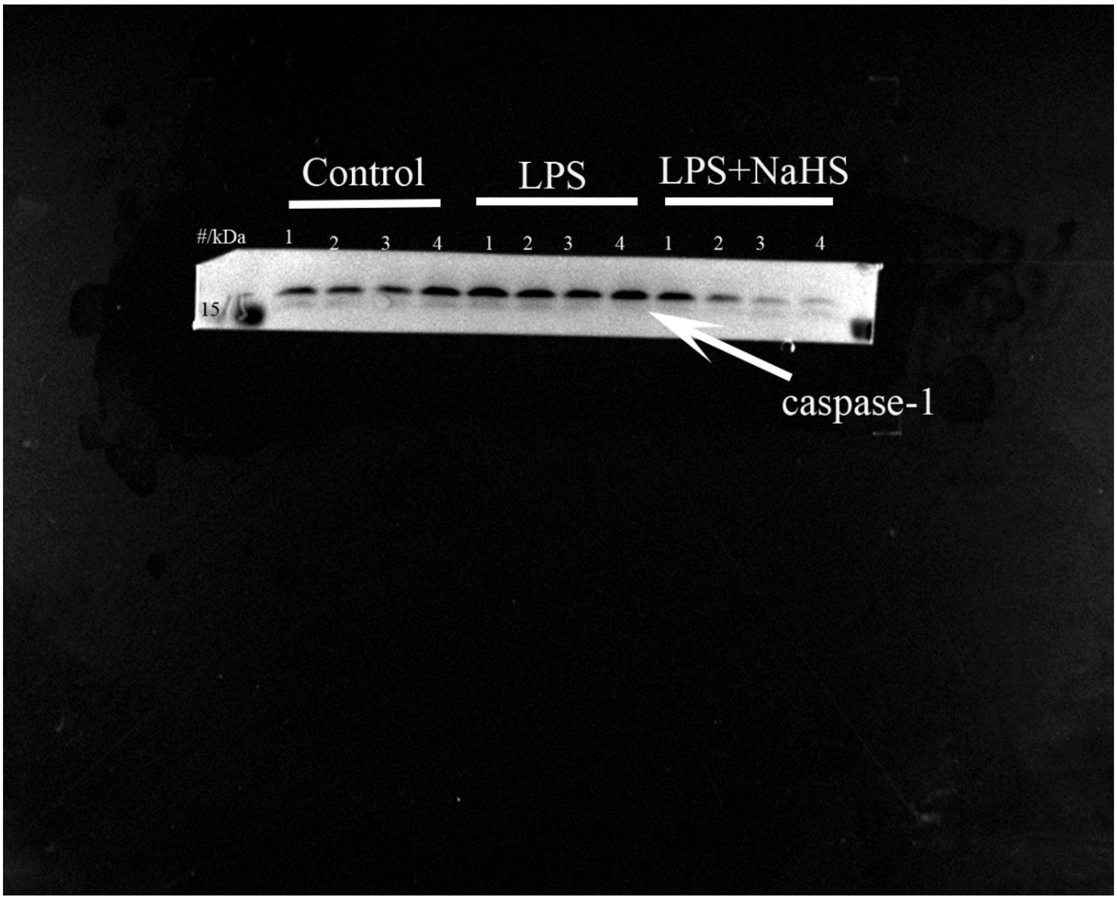

Figure S9. Western blot membrane of caspase-1 (~20 kDa) protein detected with anti-caspase-1 (A0964; 1:1000; ABclonal, China) antibody. Gel-separated proteins were transferred to PVDF membranes (0.2 μm pore size; Millipore, Bedford, MA) via wet electroblotting (200mA, 30 min). Membranes, incubated with a horseradish peroxidase-conjugated secondary antibody (AS014; 1:1000; ABclonal), were developed with Omni-ECL™Femto Light Chemiluminescence Kit (Epizyme). #Weight marker (molecular weight in kDa): Multicolor Prestained Protein Ladder, 10 to 250 kDa; catalogue number: WJ102i. Blot images, prior to the densitometry readings, were converted to grayscale with ImageJ (ImageJ, National Institutes of Health, Maryland, USA) as follows: Image -> Type –> 8 bit. Next: Analyze->set scale->Click to remove scale. Then: Process->Subtract background->rolling ball radius 50 pixels-> light background. Final: Edit->invert.

Control 1, 2, 3, and 4 refer to four different samples from the control group.
LPS 1, 2, 3, and 4 show four different samples from the LPS group.
LPS+NaHS 1, 2, 3, and 4 indicate four different samples from the LPS+NaHS group.

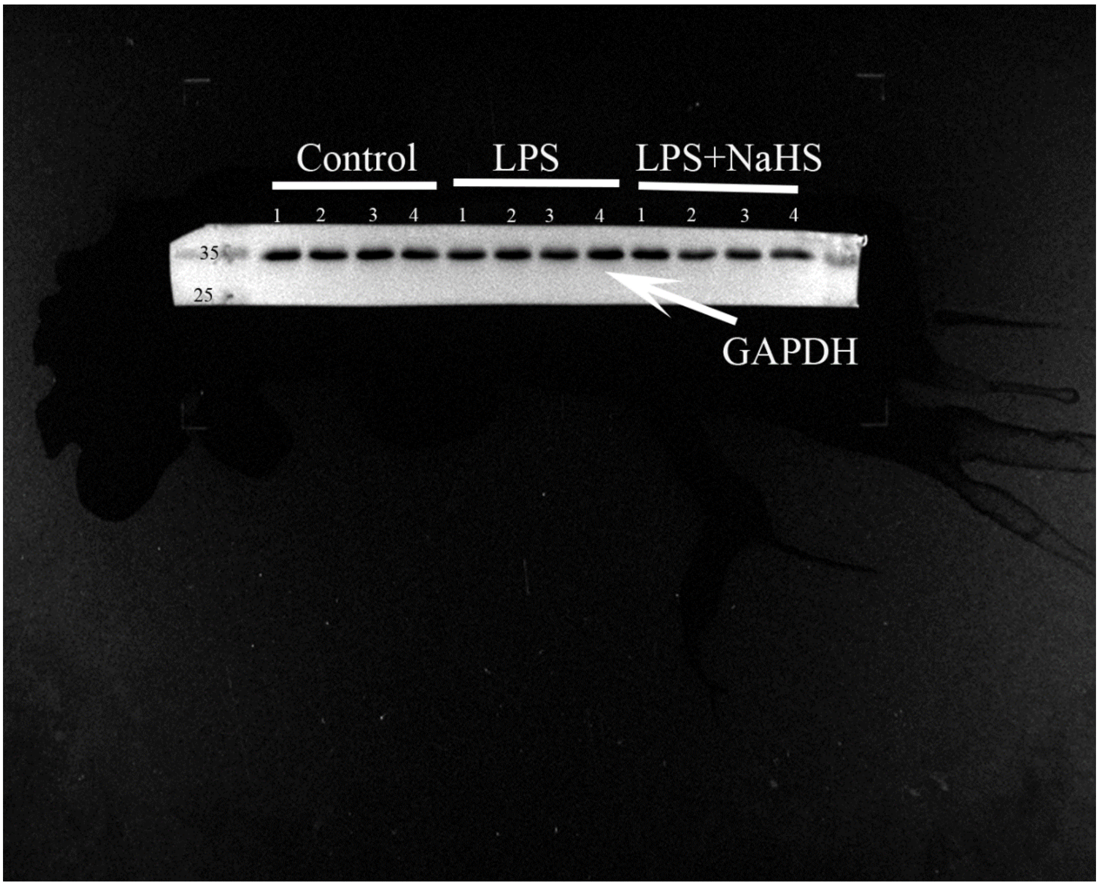

Figure S10. Western blot membrane of GAPDH (~37 kDa) protein detected with anti-GAPDH (MB001; 1:10000; Bioworld, USA) antibody. Gel-separated proteins were transferred to PVDF membranes (0.2 μm pore size; Millipore, Bedford, MA) via wet electroblotting (200mA, 30 min). Membranes, incubated with a horseradish peroxidase-conjugated secondary antibody (AS014; 1:1000; ABclonal), were developed with Omni-ECL™Femto Light Chemiluminescence Kit (Epizyme). #Weight marker (molecular weight in kDa): Multicolor Prestained Protein Ladder, 10 to 250 kDa; catalogue number: WJ102i. Blot images, prior to the densitometry readings, were converted to grayscale with ImageJ (ImageJ, National Institutes of Health, Maryland, USA) as follows: Image -> Type –> 8 bit. Next: Analyze->set scale->Click to remove scale. Then: Process->Subtract background->rolling ball radius 50 pixels-> light background. Final: Edit->invert.

Control 1, 2, 3, and 4 refer to four different samples from the control group.
LPS 1, 2, 3, and 4 show four different samples from the LPS group.
LPS+NaHS 1, 2, 3, and 4 indicate four different samples from the LPS+NaHS group.

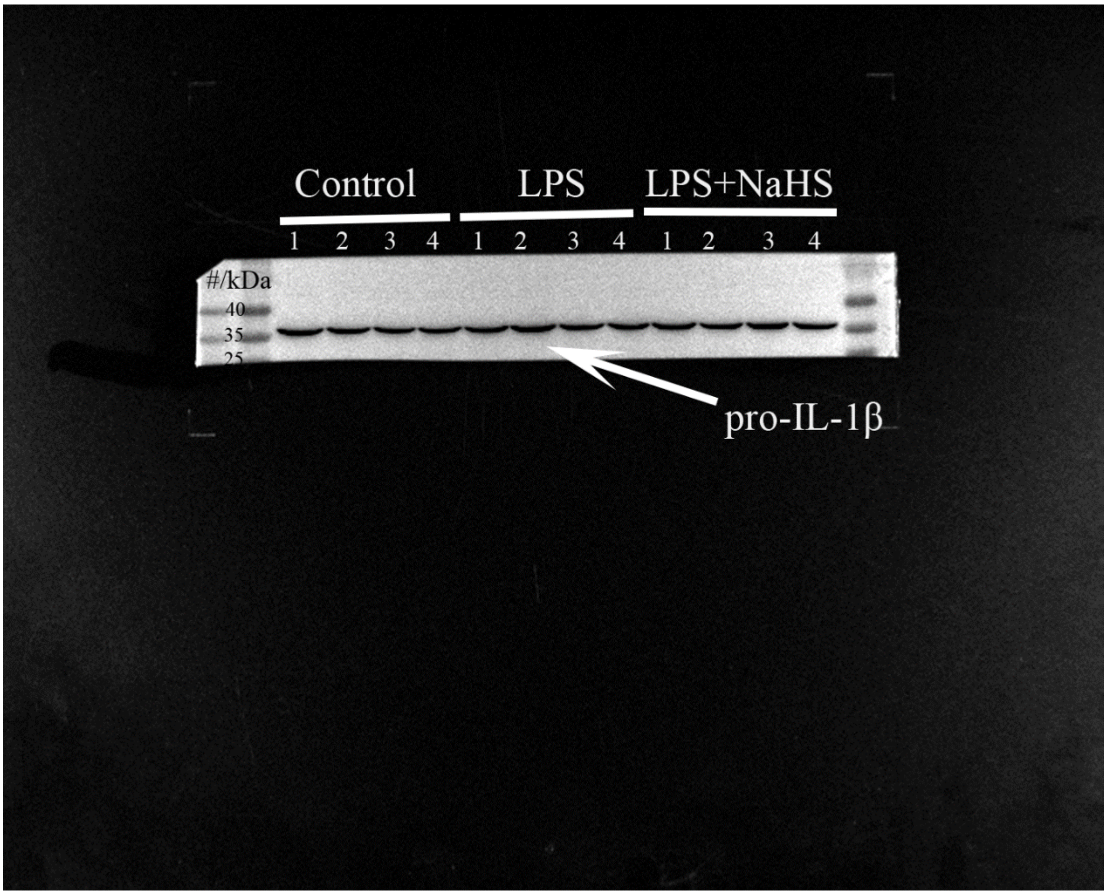

Figure S11. Western blot membrane of pro-IL-1β (~37 kDa) protein detected with anti-IL-1β (A11369; 1:1000; ABclonal, China) antibody. Gel-separated proteins were transferred to PVDF membranes (0.2 µm pore size; Millipore, Bedford, MA) via wet electroblotting (200mA, 30 min). Membranes, incubated with a horseradish peroxidase-conjugated secondary antibody (AS014; 1:1000; ABclonal), were developed with Omni-ECL™Femto Light Chemiluminescence Kit (Epizyme). #Weight marker (molecular weight in kDa): Multicolor Prestained Protein Ladder, 10 to 250 kDa; catalogue number: WJ102i. Blot images, prior to the densitometry readings, were converted to grayscale with ImageJ (ImageJ, National Institutes of Health, Maryland, USA) as follows: Image -> Type –> 8 bit. Next: Analyze->set scale->Click to remove scale. Then: Process->Subtract background->rolling ball radius 50 pixels-> light background. Final: Edit->invert.

Control 1, 2, 3, and 4 refer to four different samples from the control group.
LPS 1, 2, 3, and 4 show four different samples from the LPS group.
LPS+NaHS 1, 2, 3, and 4 indicate four different samples from the LPS+NaHS group.

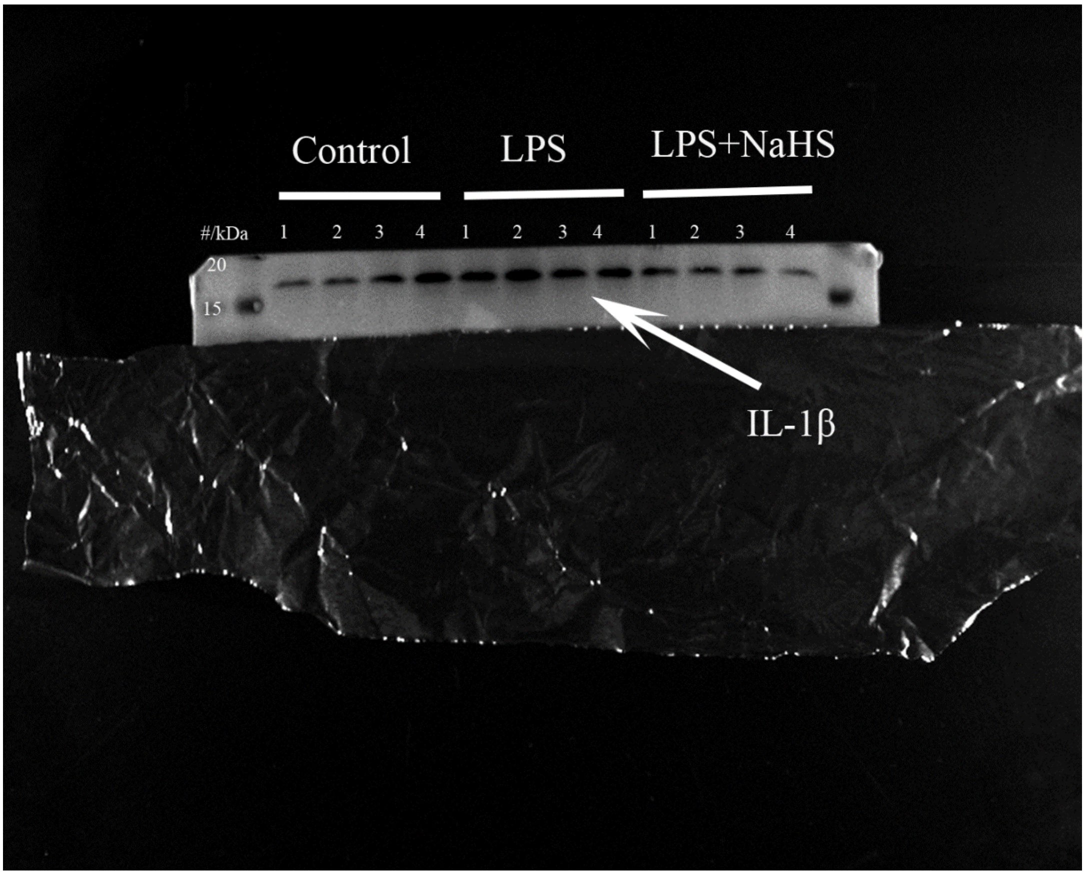

Figure S12. Western blot membrane of IL-1β (~17 kDa) protein detected with anti-IL-1β (A11369; 1:1000; ABclonal, China) antibody. Gel-separated proteins were transferred to PVDF membranes (0.2 μm pore size; Millipore, Bedford, MA) via wet electroblotting (200mA, 30 min). Membranes, incubated with a horseradish peroxidase-conjugated secondary antibody (AS014; 1:1000; ABclonal), were developed with Omni-ECL™Femto Light Chemiluminescence Kit (Epizyme). #Weight marker (molecular weight in kDa): Multicolor Prestained Protein Ladder, 10 to 250 kDa; catalogue number: WJ102i. Blot images, prior to the densitometry readings, were converted to grayscale with ImageJ (ImageJ, National Institutes of Health, Maryland, USA) as follows: Image -> Type –> 8 bit. Next: Analyze->set scale->Click to remove scale. Then: Process->Subtract background->rolling ball radius 50 pixels-> light background. Final: Edit->invert.

Control 1, 2, 3, and 4 refer to four different samples from the control group.
LPS 1, 2, 3, and 4 show four different samples from the LPS group.
LPS+NaHS 1, 2, 3, and 4 indicate four different samples from the LPS+NaHS group.

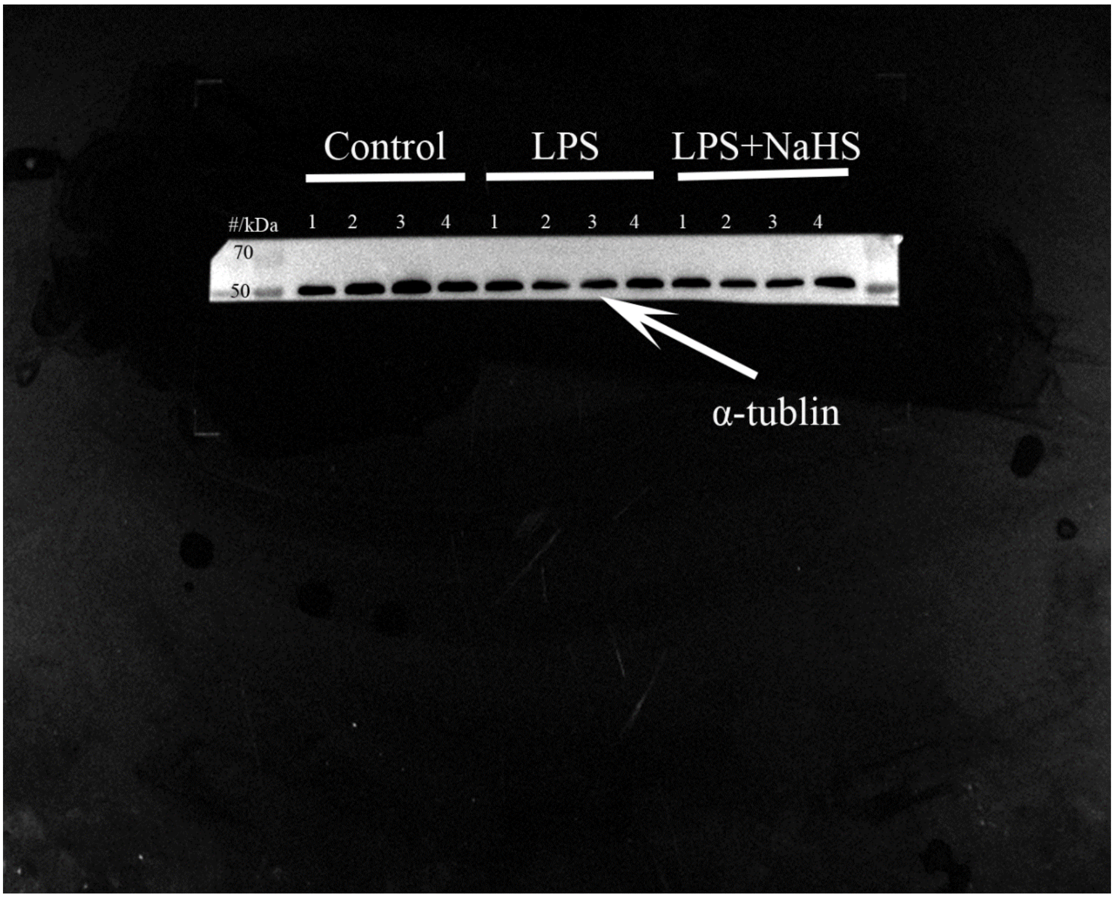

Figure S13. Western blot membrane of α-tublin (~55 kDa) protein detected with anti-α-tublin (BS1699; 1:10000; Bioworld, USA) antibody. Gel-separated proteins were transferred to PVDF membranes (0.2 μm pore size; Millipore, Bedford, MA) via wet electroblotting (200mA, 30 min). Membranes, incubated with a horseradish peroxidase-conjugated secondary antibody (AS014; 1:1000; ABclonal), were developed with Omni-ECL™Femto Light Chemiluminescence Kit (Epizyme). #Weight marker (molecular weight in kDa): Multicolor Prestained Protein Ladder, 10 to 250 kDa; catalogue number: WJ102i. Blot images, prior to the densitometry readings, were converted to grayscale with ImageJ (ImageJ, National Institutes of Health, Maryland, USA) as follows: Image -> Type –> 8 bit. Next: Analyze->set scale->Click to remove scale. Then: Process->Subtract background->rolling ball radius 50 pixels-> light background. Final: Edit->invert.

Control 1, 2, 3, and 4 refer to four different samples from the control group.
LPS 1, 2, 3, and 4 show four different samples from the LPS group.
LPS+NaHS 1, 2, 3, and 4 indicate four different samples from the LPS+NaHS group.

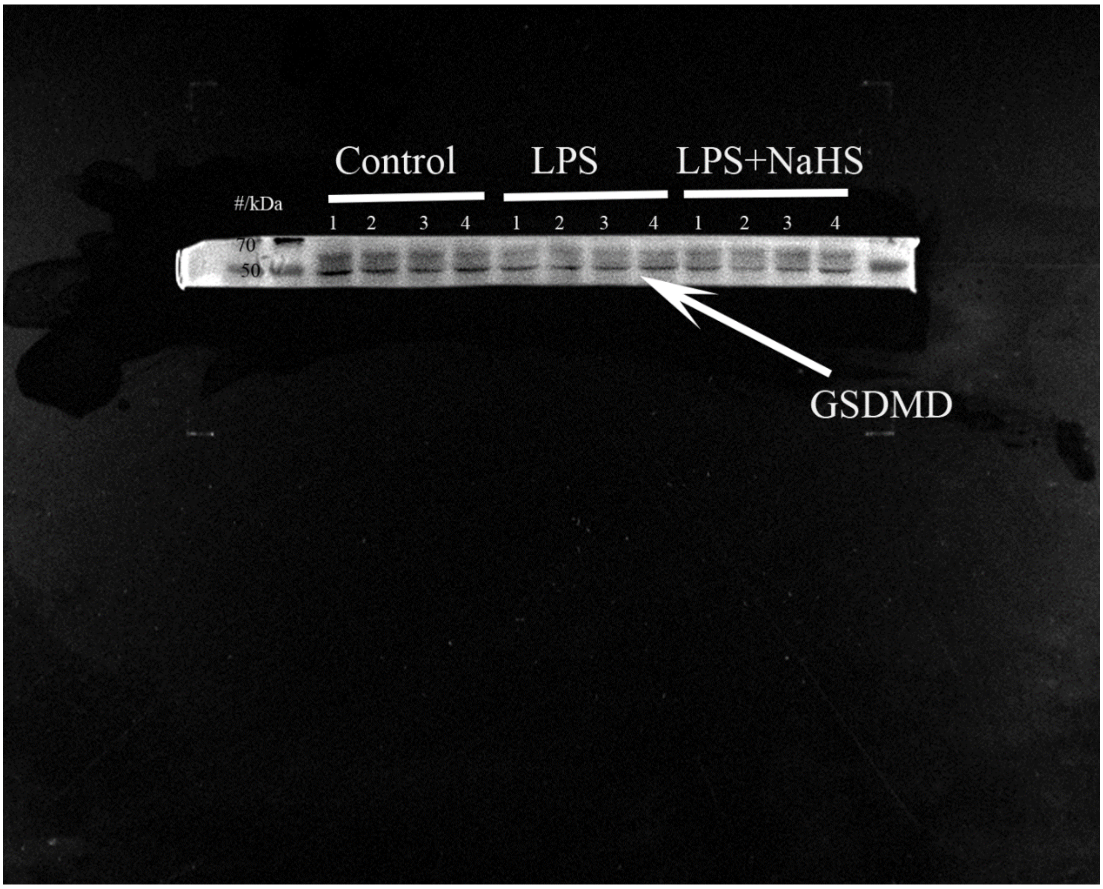

Figure S14. Western blot membrane of GSDMD (~53 kDa) protein detected with anti-GSDMD (A20197; 1:1000; ABclonal, China) antibody. Gel-separated proteins were transferred to PVDF membranes (0.2 μm pore size; Millipore, Bedford, MA) via wet electroblotting (200mA, 60 min). Membranes, incubated with a horseradish peroxidase-conjugated secondary antibody (AS014; 1:1000; ABclonal), were developed with Omni-ECL™Femto Light Chemiluminescence Kit (Epizyme). #Weight marker (molecular weight in kDa): Multicolor Prestained Protein Ladder, 10 to 250 kDa; catalogue number: WJ102i. Blot images, prior to the densitometry readings, were converted to grayscale with ImageJ (ImageJ, National Institutes of Health, Maryland, USA) as follows: Image -> Type –> 8 bit. Next: Analyze->set scale->Click to remove scale. Then: Process->Subtract background->rolling ball radius 50 pixels-> light background. Final: Edit->invert.

Control 1, 2, 3, and 4 refer to four different samples from the control group.
LPS 1, 2, 3, and 4 show four different samples from the LPS group.
LPS+NaHS 1, 2, 3, and 4 indicate four different samples from the LPS+NaHS group.

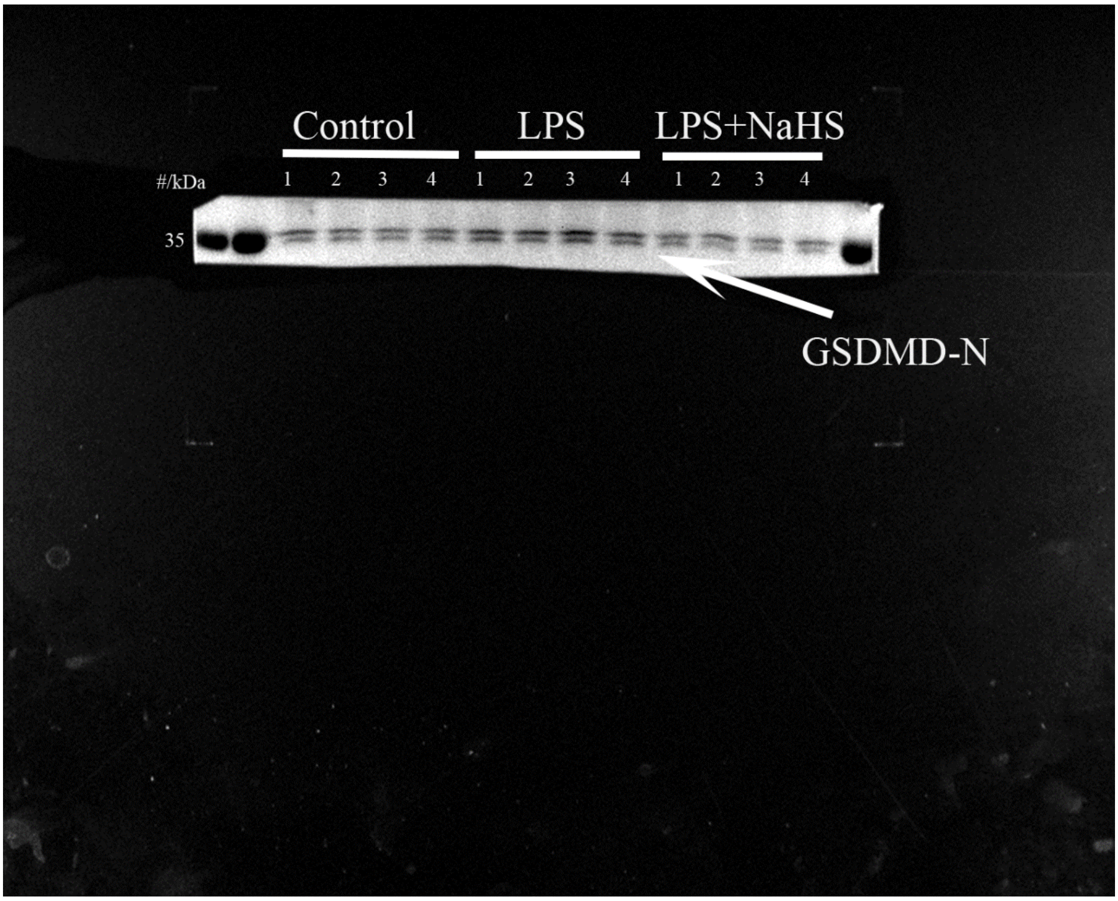

Figure S15. Western blot membrane of GSDMD-N (~37 kDa) protein detected with anti-GSDMD-N (A20197; 1:1000; ABclonal, China) antibody. Gel-separated proteins were transferred to PVDF membranes (0.2 μm pore size; Millipore, Bedford, MA) via wet electroblotting (200mA, 60 min). Membranes, incubated with a horseradish peroxidase-conjugated secondary antibody (AS014; 1:1000; ABclonal), were developed with Omni-ECL™Femto Light Chemiluminescence Kit (Epizyme). #Weight marker (molecular weight in kDa): Multicolor Prestained Protein Ladder, 10 to 250 kDa; catalogue number: WJ102i. Blot images, prior to the densitometry readings, were converted to grayscale with ImageJ (ImageJ, National Institutes of Health, Maryland, USA) as follows: Image -> Type –> 8 bit. Next: Analyze->set scale->Click to remove scale. Then: Process->Subtract background->rolling ball radius 50 pixels-> light background. Final: Edit->invert.

Control 1, 2, 3, and 4 refer to four different samples from the control group.
LPS 1, 2, 3, and 4 show four different samples from the LPS group.
LPS+NaHS 1, 2, 3, and 4 indicate four different samples from the LPS+NaHS group.

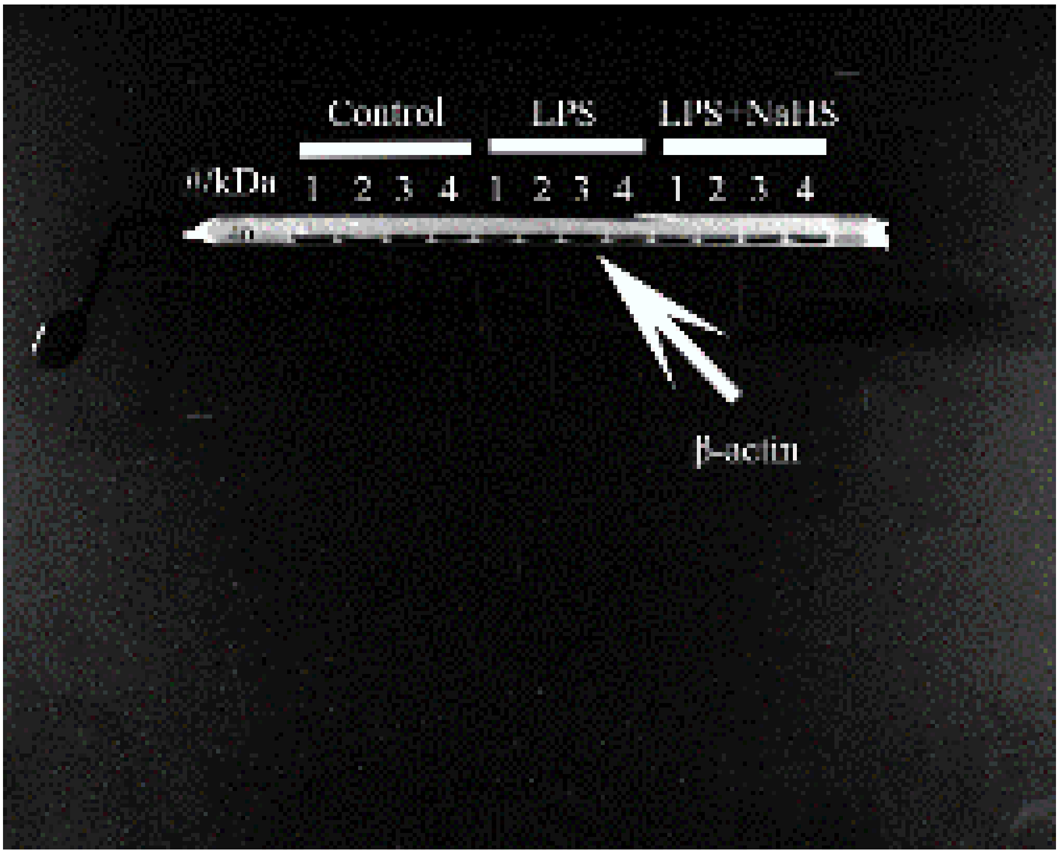

Figure S16. Western blot membrane of β-actin (~43 kDa) protein detected with anti-β-actin (BS6007M; 1:10000; Bioworld, USA) antibody. Gel-separated proteins were transferred to PVDF membranes (0.2 μm pore size; Millipore, Bedford, MA) via wet electroblotting (200mA, 60 min). Membranes, incubated with a horseradish peroxidase-conjugated secondary antibody (AS014; 1:1000; ABclonal), were developed with Omni-ECL™Femto Light Chemiluminescence Kit (Epizyme). #Weight marker (molecular weight in kDa): Multicolor Prestained Protein Ladder, 10 to 250 kDa; catalogue number: WJ102i. Blot images, prior to the densitometry readings, were converted to grayscale with ImageJ (ImageJ, National Institutes of Health, Maryland, USA) as follows: Image -> Type –> 8 bit. Next: Analyze->set scale->Click to remove scale. Then: Process->Subtract background->rolling ball radius 50 pixels-> light background. Final: Edit->invert.

Control 1, 2, 3, and 4 refer to four different samples from the control group.
LPS 1, 2, 3, and 4 show four different samples from the LPS group.
LPS+NaHS 1, 2, 3, and 4 indicate four different samples from the LPS+NaHS group.
